# Tuned Cd^2+^ Selectivity: Showcase of Electronic and Regio-Effect of π-Extended Di-2-Picolylamine-Substituted Quinoline-Based Tolans

**DOI:** 10.3390/molecules26040917

**Published:** 2021-02-09

**Authors:** Min-Sung Ko, P. Sankara Rao, Dong-Gyu Cho

**Affiliations:** Department of Chemistry and Chemical Engineering, Inha University, Inharo 100, Nam-gu, Incheon 22212, Korea; komin428@naver.com (M.-S.K.); p.sankarmsc89@gmail.com (P.S.R.)

**Keywords:** Cd^2+^-selective, turn-on fluorescence, tolan

## Abstract

π-Extended di-2-picolylamine (DPA)-substituted 8-hydroxyquinoline (8-HQ) tolans (**2**) were synthesized for testing electronic and regio-effects. The electron-poor CN-tolan (**2b**) showed clear selectivity for Cd^2+^ (>>Zn^2+^) over other metal ions via turn-on fluorescence, while the electron-rich MeO-tolan (**2a**) displayed no clear metal selectivity. Furthermore, considering that there was no significant energy difference between the Cd^2+^ complexes of **1** and **2b**, the intended regio-effect (7- vs. 5-substituted effect) did not induce steric hindrance. Thus, the regio-effect is mainly electronic. Considering the above, **2a** and **2b** constitute a complete showcase in which electronic and regio-effects modulate the metal selectivity. The fluorescence titration of **2b** (10 mM) with Cd^2+^ showed that the limit of detection (LOD) of the Cd^2+^-selective **2b** was 158 nM in PBS (phosphate-buffered saline) (10 mM, pH 7.2) containing 50% MeOH.

## 1. Introduction

**Figure 1:**
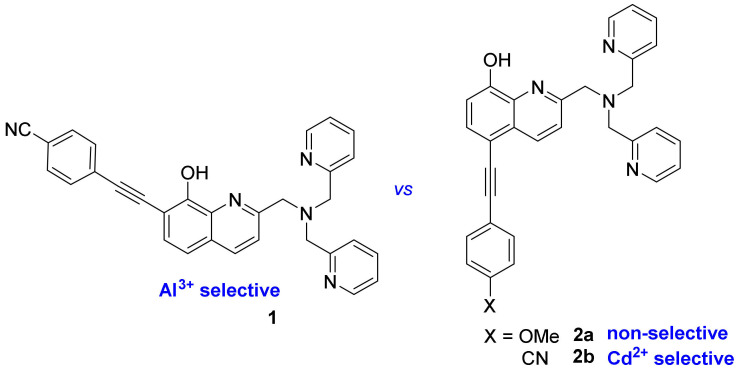
Electronic and regio-effects of a quinoline-based tolan for metal selectivity.

The introduction of aryl acetylenes into dyes is one of the most popular synthetic methods not only to extend the π-conjugation pathway of the dyes but also to induce electronic and regio-effects as a form of push-pull-type dyes [1,2]. The resultant compounds become tolan derivatives, and they have an infinite number of conformers because of the free rotation around the acetylene axis. This free rotation around the acetylene axis is a well-known characteristic of tolan derivatives. Recently, our group recognized the fluorescent dye properties of some tolan derivatives without the help of conjugation with other types of dyes. These tolans can function as switches [3,4], sensors [5,6,7], and β-turn mimics [8] depending on various installed motifs. They also exhibit a range of fluorescence quantum yields. We have also been interested in the binding properties of quinoline and its uses [9,10]. In the case of metal ion recognition, the binding motif of quinoline accelerates the Hg^2+^-catalyzed hydration of acetylene to quantify Hg^2+^ in solutions [6]. In principle, metal ion selectivity is mainly tailored by the structural characteristics of metal-binding motifs denoting preorganization and atom arrangement at the binding site. Specifically, the metal selectivity of di-2-picolylamine (DPA)-substituted 8-hydroxyquinoline (8-HQ) has been well developed for Zn^2+^ binder [11]. In principle, the compounds are poorly fluorescent because the lone pair of the tertiary nitrogen involves photoinduced electron transfer (PET), while the phenolic proton plays a role in excited-state proton transfer (ESPT). Upon specific complexation, both phenomena suddenly stop, and an enhanced fluorescence signal is detected. In the literature, although further modification of the hydroxyl group of 8-HQ did not increase the Zn^2+^ selectivity [12], our previous result showed that the selectivity can be changed to Al^3+^ over other ions, as shown in Figure 1 [13]. This change in selectivity could be due to the electronic effect of the π-extended forms of DPA-substituted-8-HQ (**1**). Thus, we further attempted to find a complete showcase that displays clear electronic (electron-withdrawing vs. electron-donating effect) and regio-effects (7- vs. 5-substitution effect) of the π-extended ethynylbenzene attached to DPA-substituted-8-HQ (**2**), as highlighted in Figure 1. Herein, we demonstrate these two effects (electronic and regio-effects) that allow **2b** to achieve Cd^2+^ selectivity over other ions, along with π-extension from 8-HQ. Receptor **2b** shows an increased fluorescent band at 557 nm upon the addition of Cd^2+^. It should be noted that DPA-substituted-8-HQ is fluorescence silent [12]. In addition, Cd^2+^ is a highly toxic heavy metal ion that is nonbiodegradable and has a long elimination half-life (10–30 years) in the human body [14]. Thus, the maximum allowed Cd^2+^ concentration in drinking water containing microgram per liter has been recommended by the U.S. Environmental Protection Agency (EPA) [15]. In principle, various analytical techniques such as atomic absorption/emission spectroscopy [16,17], electrochemical methods [18], and inductively coupled plasma-mass spectrometry (ICP-MS) have been used for the determination of Cd^2+^. In contrast, the use of small-molecule fluorescent receptors has several advantages over other methods, such as high sensitivity, easy sample preparation, rapid response, and application at the cellular level [19,20]. Thus, Cd^2+^-selective fluorescent receptors (chemosensors) have been developed [21,22,23,24,25].

## 2. Results and Discussion

### 2.1. Synthesis

**Scheme 1:**
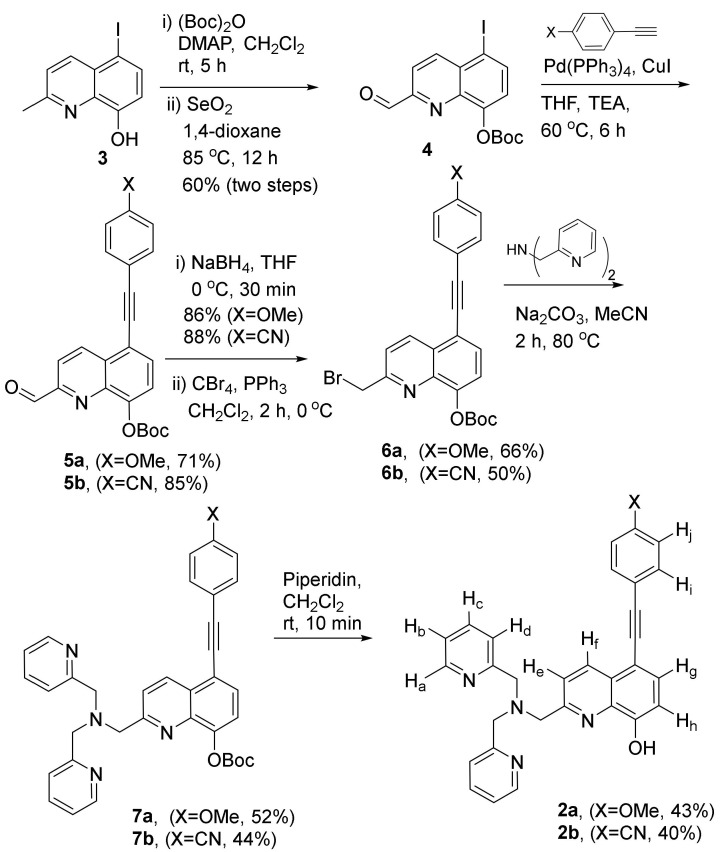
Synthetic scheme for the preparation of quinoline-based tolan (**2**).

To test the proposed concepts, **2a** and **2b** were designed and prepared from 5-iodo-2-methylquinolin-8-ol as the starting material by modifying our previous synthetic process depicted in Scheme 1 [13]. Allylic oxidation of the methyl group of **3** was attempted, and the product was obtained in 60% yield via two consecutive reactions, where *tert*-butyloxycarbonyl (BOC) protection was followed by SeO_2_ oxidation. The obtained product (**4**) was π-extended with phenylacetylene via Sonogashira coupling. The π-extended aldehyde was reduced with NaBH_4_ and then brominated in good yield. The brominated compound **6** was alkylated with di-2-picolylamine in moderate yields. The final target compounds were obtained in moderate yields by deprotection of the BOC group.

### 2.2. Cation-Binding Studies

**Figure 2:**
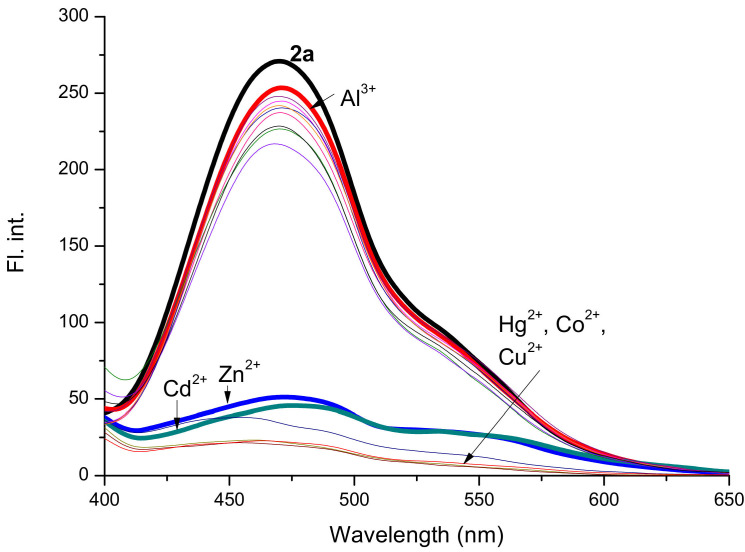
Fluorescence spectra of **2a** (10 μM) recorded with or without 2 equiv. of metal ions (Al^3+^, Zn^2+^, Cd^2+^, Ag^+^, Co^2+^, Ni^2+^, Cu^2+^, K^+^, Pb^2+^, Mg^2+^, Na^+^, Ca^2+^, Fe^2+^, Fe^3+^, and Hg^2+^) in PBS buffer (10 mM, pH 7.2) containing 50% MeOH.

**Figure 3:**
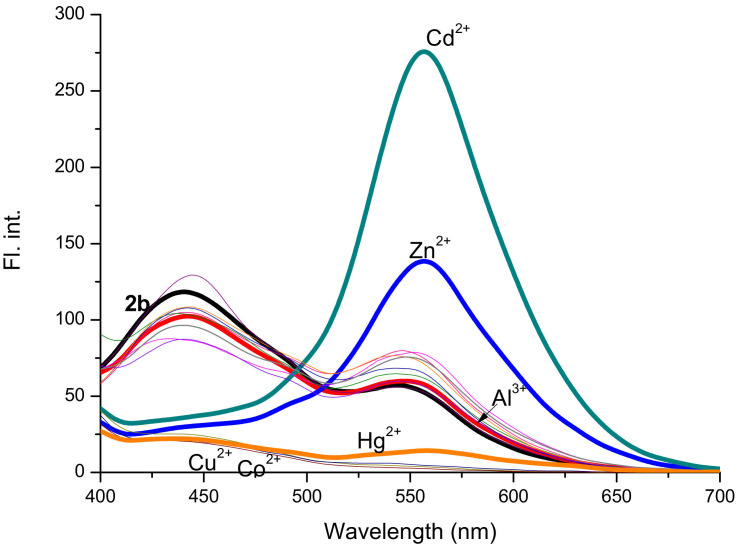
Fluorescence spectra of **2b** (10 μM) recorded with or without 2 equiv. of metal ions (Al^3+^, Zn^2+^, Cd^2+^, Ag^+^, Co^2+^, Ni^2+^, Cu^2+^, K^+^, Pb^2+^, Mg^2+^, Na^+^, Ca^2+^, Fe^2+^, Fe^3+^, and Hg^2+^) in PBS buffer (10 mM, pH 7.2) containing 50% MeOH.

To rapidly evaluate the metal selectivity, the tolans (**2**, 10 μM) were titrated with various metal ions (2 equiv. of each) in PBS (10 mM, pH 7.2) containing 50% MeOH. The previous 7-substituted quinoline (**1**) exhibited “turn on” fluorescence for Al^3+^ (Figure 1). Interestingly, **2a** displayed “turn off” fluorescence for Zn^2+^, Cd^2+^, Ni^2+^, Co^2+^, and Hg^2+^, while its fluorescence changes were not significant for other metal ions (Figure 2). In contrast, **2b** exhibited Cd^2+^ selectivity over Al^3+^ (Figure 3). These results support the idea that the electronic and regio-effects of the designed metal ion receptors endow specific metal ion selectivity without changing the metal-binding motifs. To explain the different metal selectivities of the tolans (**1**, **2a**, and **2b**), electrostatic potential maps were calculated based on the density functional theory (DFT; B3LYP/6-31G**, Figure S8 in the ESI). As expected, the highest electron density of the quinoline nitrogen atoms was found in **2a**, followed by **2b** and **1** (**2a** >> **2b** > **1**). Based on the hard and soft acids and bases (HSAB) theory, Al^3+^, Zn^2+^, and Cd^2+^ were classified as hard, intermediate, and soft Lewis acids, respectively. The reported Al^3+^ selectivity of **1**, which is regarded as the hardest ligand, can be explained by this theory [13]. Similarly, the Cd^2+^ (>>Zn^2+^) selectivity of **2b** can be partly explained by the increased electron density of the quinoline nitrogen atom. We assumed that **2a** could be unselective to most of the metal ions because of its electron-rich nature. In addition, steric hindrance (**2b** vs. **1**) resulting from the regio-effect is not observed because of the nearly same energy levels of the optimized structures of [Cd^2+^(**2b**-H)]^+^ and [Cd^2+^(**1**-H)]^+^ (Tables S1 and S2 in the ESI). Thus, we concluded that the observed regio-effect of tolan is related to the electronic effect in this system.

**Figure 4:**
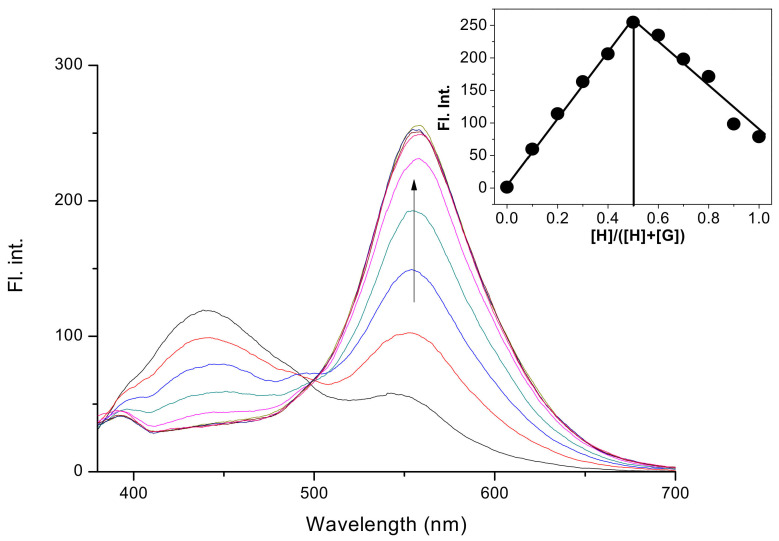
Fluorescence titration of **2b** (10 μM) recorded with various amounts of Cd^2+^ (0 to 1.6 equiv.). Inset: Job plot analysis of the interaction between **2b** and Cd^2+^.

The UV–VIS spectrum of CN-tolan (**2b**) featured λ_max_ bands at 356 nm and a long tail to 500 nm (Figure S5 in the ESI). Upon gradual addition of Cd^2+^, the intensity of the shoulder at 404 nm increased and saturated at 1 equiv. of Cd^2+^. Isosbestic points were found at 286 and 368 nm, suggesting a 1:1 binding between Cd^2+^ and **2b**. Additionally, the fluorescence titration of **2b** with Cd^2+^ in PBS buffer (10 mM, pH 7.2) containing 50% MeOH was performed, and fluorescence enhancement was observed (Figure 4). In particular, the fluorescence intensity increased approximately fivefold at 559 nm as the concentration of Cd^2+^ increased from 0 to 1.6 equiv. with respect to **2b**. The binding of Cd^2+^ appeared strong, as judged from the fact that only 1 equiv. of Cd^2+^ was required to achieve saturation. The binding data were analyzed using the Hill equation:  log(*Y*/(*1* − *Y*)) = *n* log[Cd^2+^] + log*K*, where *Y* is the fractional saturation of the host, *n* is the Hill coefficient, and *K* is the association constant. From the intercept and slope of the linear part of the plot, we obtained log*K* = 11.3 ± 0.3 and *n* = 2.1 ± 0.06, with a correlation coefficient of 0.99 (Figures S2 and S3 in ESI). The values were quite similar to those obtained in the UV–VIS titration (log*K* = 10.6 ± 0.3 and *n* = 2.0 ± 0.06, with a correlation coefficient of 0.99, Figures S6 and S7 in ESI). These similar values obtained from two different titration methods supported the validity of the constants. In addition, the limit of detection (LOD) for Cd^2+^ was calculated using the 3σ method (σ = 1.039, m = 1.97 × 10^7^ M^−1^) [7] and found to be 158 nM (Figure S4 in the ESI).

To further investigate the binding mode of Cd^2+^ with **2b,** ^1^H NMR titration of **2b** (2.0 mM) was performed with Cd^2+^ in CD_3_CN (Figure S9 in the ESI). In the absence of Cd^2+^, the ^1^H NMR signals of H_c_, H_e_, H_g_, H_i_, and H_j_ partially overlapped in the spectrum (Scheme 1). Upon the addition of 0.4 equiv. of Cd^2+^, most of the **2b** signals broadened; then, they were well resolved and saturated at 0.8 equiv. of Cd^2+^ (Figure S9 in the ESI). The signals of H_i_ and H_j_ appeared as a singlet even after the addition of 1.6 equiv. of Cd^2+^. These signals of the synthetic precursors were not resolved because they were AA’BB’ type spin systems. These NMR traits supported the fact that all the heteroatoms of **2b** participated in Cd^2+^ binding. All the protons were assigned accurately in the 2D NMR spectrum of **2b**, while the H_g_ and H_h_ signals were assigned by comparing their relative chemical shifts (Figures S10 and S11 in the ESI).

### 2.3. ESI-MS Studies and Theoretical Calculations

**Figure 5:**
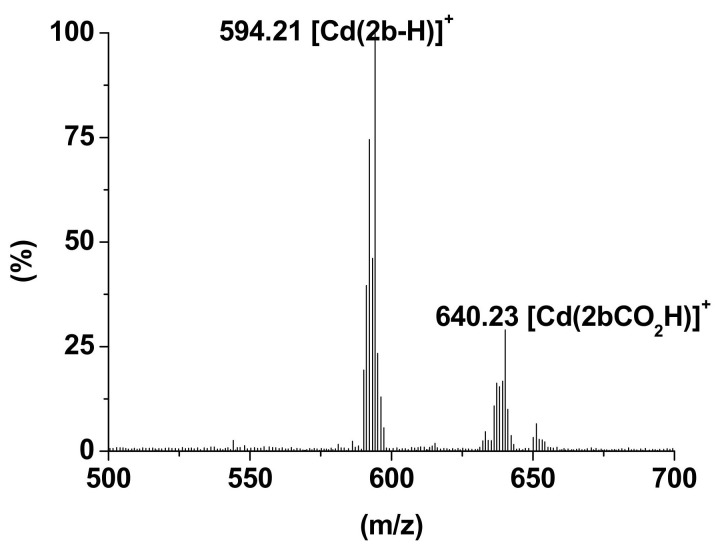
Cd^2+^ complex of tolan (**2b**) found from the positive ion mode electrospray ionization-mass spectrometry (ESI-MS) spectrum.

**Figure 6:**
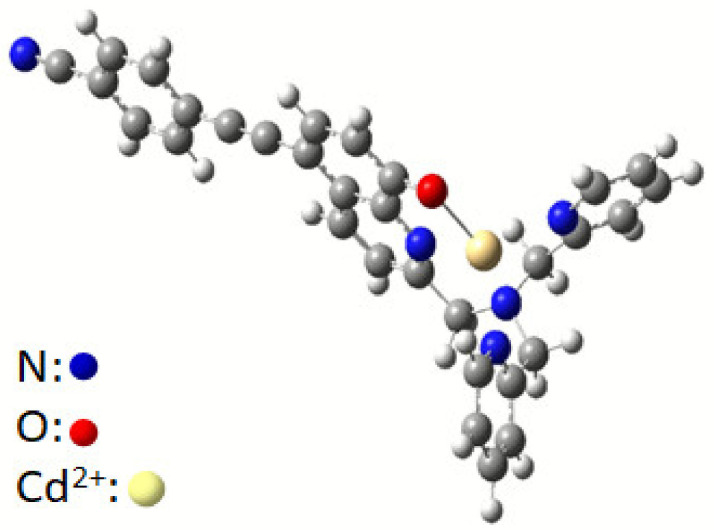
Calculated structure of the Cd^2+^ complex of **2b** ([Cd(**2b**-H)]^+^) based on density functional theory (B3LYP/6-31G**).

A 1:1 stoichiometry of the Cd^2+^ complex with tolan was expected from the isosbestic points obtained from the UV–VIS titration (Figure S5 in the ESI). Job plot analysis supported the stoichiometry by the fluorescence method (inset of Figure 4). Additionally, a possible complex with Cd^2+^ was detected by electrospray ionization-mass spectrometry (ESI-MS; calculated *m/z* for [Cd(**2b**-H)]^+^: 594.09; found: 594.21) using CH_3_CN containing 0.1% HCO_2_H (Figure 5) [26]. To understand the binding mode of **2b**, theoretical calculations were conducted based on information obtained from the ESI mass data at the B3LYP/6-31G** level of theory (Figure 6). In the optimized structure, the extended tolan structure was far from the Cd^2+^ binding site, as expected. This information also indicated that the observed change in metal selectivity is derived from the electronic effect.

### 2.4. Cd^2+^ Selectivity of **2b**

**Figure 7:**
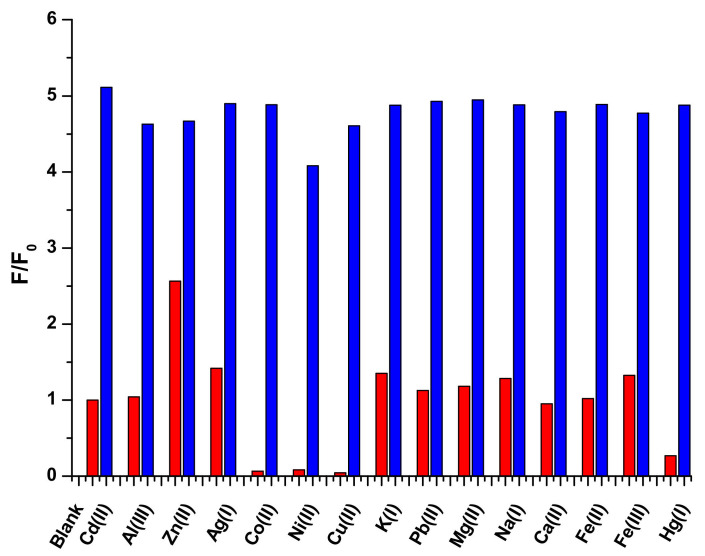
Metal-ion selectivity of **2b** (10 μM) in PBS buffer (10 mM, pH 7.2) containing 50% MeOH. The red bars represent the F/F_0_ values for **2b** + 2 equiv. of each metal ion, whereas the blue bars represent F/F_0_ values for **2b** + 2 equiv. of each metal ion + 2 equiv. of Cd^2+^; F_0_: emission intensity of **2b**; F: emission intensity of **2b** in the presence of 2 equiv. of each cation or each cation + Cd^2+^.

**Figure 8:**
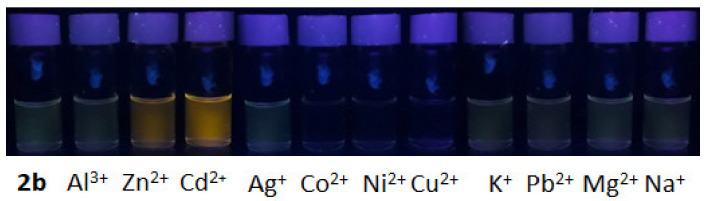
Change in the fluorescence of tolan (**2b**, 10 μM) in PBS buffer (10 mM, pH 7.2) containing 50% MeOH, 10 min after the addition of each metal (2 equiv.) under a laboratory hand-held UV lamp. Three individual photos were combined.

The selectivity of **2b** was further investigated in PBS buffered solutions. The presence of other metal ions slightly interfered with the detection of Cd^2+^ (Figure 7). However, the metal selectivity of CN-tolan (**2b**) was selective for Cd^2+^ and altered compared to MeO-tolan (**2a**). Further experiments also demonstrated that **2b** can be used as an indicator under a laboratory hand-held UV lamp for Cd^2+^ and Zn^2+^ detection by the naked eye or with the aid of a fluorometer (Figure 8). The solution colors of **2b** with Zn^2+^ or Cd^2+^ showed only a subtle difference. The difference could be monitored by the two intensity ratios in the presence of Cd^2+^ (I_555_ /I_500_ = 3.80: fluorescence intensity ratio of 555 and 500 nm) and Zn^2+^ (I_555_ /I_500_ = 2.82). In the past studies, the differentiation of Cd^2+^ from Zn^2+^ was a challenge due to their similar chemical properties [12,27]. In addition, the slightly different fluorescence spectrum of **2a** (I_468_/I_505_: intensity ratio of 468 and 505 nm) can also allow the differentiation of Cd^2+^ from Zn^2+^ in PBS buffered solution.

## 3. Materials and Methods

### 3.1. Materials and Synthesis

Reagents were purchased at the highest commercial quality and used without further purification, unless otherwise stated. The yields of the synthesized compounds were measured after chromatographic purification.

The syntheses of all the new compounds are summarized in Scheme 1. All new compounds are characterized by standard methods, including ^1^H NMR, ^13^C NMR, and high-resolution mass spectroscopy. The ^1^H and ^13^C NMR spectra are included in the ESI.

### 3.2. Fluorescence and UV–VIS Titrations

Upon the addition of incremental amounts of Cd^2+^ to the solution of **2b** (from 0 to 1.8 equiv. of Cd^2+^), the absorbance or fluorescence change of **2b** (10 μM) was recorded in PBS buffer (10 mM, pH 7.2) containing 50% MeOH. The equilibrium constants of the complexes were calculated using the Hill equation:  log(*Y*/(*1 − Y*)) = *n* log[Cd^2+^] + log*K*, where *Y* is the fractional saturation of the host, *n* is the Hill coefficient, and *K* is the association constant. All these data are included in the ESI.

### 3.3. Theoretical Calculations

All density functional theory (DFT) calculations were performed using a Gaussian 09 program package. Calculations for structural optimizations and frequency calculations were carried out using the DFT method with Becke’s three-parameter hybrid exchange functionals and the Lee-Yang-Parr correlation functional (B3LYP) employing the 631LAN basis set. The 631LAN basis set is composed of 6-31G** for carbon, hydrogen, nitrogen, and oxygen, and LANL2DZ for Cd^2+^.

## 4. Conclusions

To test the electronic and regio-effects, DPA-substituted-8-HQ tolans (**2**) were synthesized. The electron-poor CN-tolan (**2b**) exhibited clear Cd^2+^ (>> Zn^2+^) selectivity, while electron-rich MeO-tolan (**2a**) displayed no clear metal selectivity. The intended regio-effect (7- vs. 5-substitution effect) related to steric hindrance was not observed because there was no significant energy difference between the metal complexes of **1** and **2b**. Thus, although the observed regio-effect contributes to metal selectivity, the contribution mainly comes from electronic effects. Considering the above, **2a** and **2b** should constitute a unique and rare showcase in which electronic and regio-effects modulate the metal selectivity. The fluorescence titration of **2b** (10 μM) was conducted with Cd^2+^ in PBS buffer (10 mM, pH 7.2) containing 50% MeOH, and the LOD of the Cd^2+^-selective **2b** was calculated to be 158 nM. However, the LOD of **2b** should be improved using a better commercial fluorometer.

## Data Availability

The data presented in this study are available in the article and suplementary materials.

## Supplementary Materials

General procedures, synthesis, and characterization of all new compounds, titration studies, ^1^H NMR binding experiments, and DFT calculations are available online.
