# Supplementary material for: Tuned Cd2+ Selectivity: Showcase of Electronic and Regio-Effect of π-Extended Di-2-Picolylamine-Substituted Quinoline-Based Tolans
Source: Molecules. 2021 Feb 9;26(4):917. doi: 10.3390/molecules26040917 (PMC7916104; doi:10.3390/molecules26040917)
Supplement: Supplementary file 1 [file molecules-26-00917-s001.pdf]

Supporting Information

Tuned Cd^{2+} Selectivity: Showcase of Electronic and Regio-effect of π-extended Di-2-picolylamine-substituted Quinoline-based Tolans

Min-Sung Ko, P. Sankara Rao, and Dong-Gyu Cho*

Department of Chemistry and Chemical Engineering, Inha University, Inharo 100, Namgu, Incheon, 22212, Republic of Korea

E-mail: dgcho@inha.ac.kr

Contents

General experimental and synthetic details **S2**

Binding Studies **S5**

DFT Calculations **S9**

NMR Studies **S13**

NMR Spectra **S15**

General experimental and synthetic details

Reagents were purchased at the highest commercial quality and used without further purification, unless otherwise stated. Yields of synthesized compounds were measured after chromatographic purification. ^{1}H and ^{13}C-NMR spectra were measured at 25 ºC using 400 MHz spectrometers. HRMS were recorded by EI or FAB.

***tert*-butyl 2-formyl-5-iodoquinolin-8-yl carbonate** (**4**)
5-iodo-2-methylquinolin-8-ol (530 mg, 1.86 mmol) was dissolved in DCM (10 mL). DMAP and $(Boc)_2O$ (447 mg, 2.05 mmol) were added to the solution and stirred for 5 hours. The solution was filtered and the filtrate was concentrated under reduced pressure. The residue was purified with short silica gel pad to afford *tert*-butyl (5-iodo-2-methylquinolin-8-yl) carbonate. The obtained product was dissolved in dioxane and SeO_2 (331 mg, 3.01 mmol) was added to the solution. The reaction was stirred and heated to 85 ºC for 12 hours. After cooling the reaction to room temperature, its solvent was evaporated and the residue was extracted with CH_2Cl_2/water. The combined organic layers were collected, dried over anhydrous Na_2SO_4, filtered, and concentrated under reduced pressure. The residue was purified over silica gel to afford **4** (445 mg, 60%).
^{1}H NMR (400 MHz, $CDCl_3$) δ 10.19 (d, *J* = 0.9 Hz, 1H), 8.51 (dd, *J* = 8.7, 0.9 Hz, 1H), 8.21 (d, *J* = 8.1 Hz, 1H), 8.08 (d, *J* = 8.7 Hz, 1H), 7.36 (d, *J* = 8.1 Hz, 1H), 1.60 (s, 9H); ^{13}C NMR (100 MHz, $CDCl_3$) δ 192.61, 152.58, 151.43, 149.02, 142.30, 141.65, 139.75, 132.81, 123.17, 119.47, 94.40, 84.37, 27.74; HRMS–FAB: m/z $[M + H]^+$ calcd for $C_{15}H_{15}INO_4$: 400.0046; found:400.0048.

***tert*-butyl 2-formyl-5-((4-methoxyphenyl)ethynyl)quinolin-8-yl carbonate** (**5a**)
1-ethynyl-4-methoxybenzene (66 mg, 0.50 mmol), $Pd(PPh_3)_4$ (34.7 mg, 0.06 equiv.), and CuI (5.7 mg, 0.06 equiv.) were added to a well-stirred solution of **4** (200 mg, 0.50 mmol) in dry THF (3 mL) and TEA (0.3 mL). The resulting mixture was heated at 60 °C for 5 hours. After cooling to room temperature, the reaction mixture was extracted with EtOAc/water. The combined organic layers were dried over anhydrous Na_2SO_4 and then filtered. The filtrate was concentrated under reduced pressure to obtain the residue. The residue was purified over silica gel to afford **5a** (14 mg, 71%).
^{1}H NMR (400 MHz, $CDCl_3$) δ 10.18 (d, *J* = 0.9 Hz, 1H), 8.85 (dd, *J* = 8.6, 0.9 Hz, 1H), 8.12 (d, *J* = 8.6 Hz, 1H), 7.86 (d, *J* = 7.9 Hz, 1H), 7.67 – 7.39 (m, 3H), 6.93 (d, *J* = 8.9 Hz, 2H), 3.85 (s, 3H), 1.62 (s, 9H); ^{13}C NMR (100 MHz, $CDCl_3$) δ 193.32, 160.32, 152.36, 151.59, 147.94, 140.99, 136.67, 133.38, 132.51, 131.40, 121.70, 120.21, 118.33, 114.57, 114.31, 95.88, 84.25, 84.01, 55.47, 27.78; HRMS–FAB: m/z $[M + H]^+$ calcd for $C_{24}H_{22}NO_5$: 404.1498; found:404.1501.

***tert*-butyl 5-((4-cyanophenyl)ethynyl)-2-(formylmethyl)quinolin-8-yl carbonate** (**5b**)
4-ethynylbenzonitrile (63 mg, 0.50 mmol), $Pd(PPh_3)_4$ (34.6 mg, 0.06 equiv.), and CuI (5.7 mg, 0.06 equiv.) were added to a well-stirred solution of **4** (200 mg, 0.50 mol) in dry THF (3 mL) and TEA (0.3 mL). The resulting mixture was heated at 60 °C for 5 hours. After cooling to room temperature, the reaction mixture was extracted with EtOAc/water. The combined organic layers were dried over anhydrous Na_2SO_4 and then filtered. The filtrate was concentrated under reduced pressure to obtain the residue. The residue was purified over silica gel to afford **5b** (169 mg, 85%).
^{1}H NMR (400 MHz, $CDCl_3$) δ 10.19 (d, *J* = 0.9 Hz, 1H), 8.80 (dd, *J* = 8.6, 0.9 Hz, 1H), 8.15 (d, *J* = 8.6 Hz, 1H), 7.93 (d, *J* = 7.9 Hz, 1H), 7.75 – 7.65 (m, 4H), 7.61 (d, *J* = 7.9 Hz, 1H), 1.63 (s, 9H); ^{13}C NMR (100 MHz, $CDCl_3$) δ 193.04, 152.52, 151.43, 149.01, 140.97, 136.23, 133.61, 132.33, 131.37, 127.35, 121.74, 118.70, 118.59, 118.37, 112.40, 93.66, 89.28, 84.51, 27.77; HRMS–FAB: m/z $[M + H]^+$ calcd for $C_{24}H_{19}N_2O_4$: 399.1345; found:399.1347.

2-(bromomethyl)-5-((4-methoxyphenyl)ethynyl)quinolin-8-yl *tert*-butyl carbonate (**6a**)
$NaBH_4$ (55 mg, 1.48 mmol) was added in portion to a well-stirred solution of **5a** (150 mg, 0.37 mmol) in THF (3 mL) at 0 ºC. The reaction mixture was slowly warmed to room temperature and stirred for 30

min. The reaction mixture was quenched with saturated NH_4Cl and the solvent was evaporated under reduced pressure. The crude product was diluted with EtOAc (8 mL), washed with water and dried over sodium sulphate and it was evaporated under reduced pressure to give alcohol compound. Without further purification, alcohol compound was diluted with CH_2Cl_2 (4 mL) and triphenylphosphine (125 mg, 0.477 mmol) and tetrabromomethane (179 mg, 0.541 mmol) were added at 0 °C. The mixture was stirred at the same temperature for 2 h. After concentration in vacuo, the residue was purified by flash column chromatography to give **6a** (114 mg, 66%).

^{1}H NMR (400 MHz, $CDCl_3$) δ 8.69 (d, *J* = 8.7 Hz, 1H), 7.72 (d, *J* = 7.9 Hz, 1H), 7.68 (d, *J* = 8.7 Hz, 1H), 7.55 (d, *J* = 8.9 Hz, 2H), 7.47 (d, *J* = 7.9 Hz, 1H), 6.92 (d, *J* = 8.9 Hz, 2H), 4.68 (s, 2H), 3.85 (s, 3H), 1.60 (s, 9H); ^{13}C NMR (100 MHz, $CDCl_3$) δ 160.15, 157.28, 151.68, 147.42, 140.28, 136.21, 133.29, 130.43, 128.84, 122.37, 121.12, 119.69, 114.89, 114.27, 95.15, 84.46, 83.90, 55.46, 34.08, 27.78; HRMS–FAB: m/z $[M + H]^+$ calcd for $C_{24}H_{23}BrNO_4$: 468.0811; found:468.0813.

2-(bromomethyl)-5-((4-cyanophenyl)ethynyl)quinolin-8-yl tert-butyl carbonate (6b)

$NaBH_4$ (56 mg, 1.51 mmol) was added in portion to a well-stirred solution of **5b** (150 mg, 0.37 mmol) in THF (3 mL) at 0 °C. The reaction mixture was slowly warmed to room temperature and stirred for 30 min. The reaction mixture was quenched with saturated NH_4Cl and the solvent was evaporated under reduced pressure. The crude product was diluted with EtOAc (8 mL), washed with water and dried over sodium sulphate and it was evaporated under reduced pressure to give alcohol compound. Without further purification, alcohol compound was diluted with CH_2Cl_2 (4 mL) and triphenylphosphine (130 mg, 0.496 mmol) and tetrabromomethane (186 mg, 0.562 mmol) were added at 0 °C. The mixture was stirred at the same temperature for 2 h. After concentration in vacuo, the residue was purified by flash column chromatography to give **6b** (86 mg, 50%).

^{1}H NMR (400 MHz, $CDCl_3$) δ 8.63 (d, *J* = 8.7 Hz, 1H), 7.78 (d, *J* = 7.9 Hz, 1H), 7.71 (d, *J* = 8.7 Hz, 1H), 7.69 (s, 4H), 7.50 (d, *J* = 7.9 Hz, 1H), 4.68 (s, 2H), 1.61 (s, 9H); ^{13}C NMR (100 MHz, $CDCl_3$) δ 157.64, 151.52, 148.48, 140.28, 135.74, 132.29, 132.25, 131.51, 128.83, 127.67, 122.78, 121.17, 118.44, 118.10, 112.16, 93.08, 89.90, 84.14, 33.89, 27.77; HRMS–FAB: m/z $[M + H]^+$ calcd for $C_{24}H_{20}BrN_2O_3$: 463.0657; found:463.0661.

2-((bis(pyridin-2-ylmethyl)amino)methyl)-5-((4-methoxyphenyl)ethynyl)quinolin-8-yl *tert*-butyl carbonate (7a)

Na_2CO_3 (14 mg, 0.13 mmol) was added to a well-stirred solution of **6a** (70 mg, 0.15 mmol) and bis(pyridin-2-ylmethyl)amine (23 mg, 0.12 mol) in CH_3CN (6 mL) at rt. The resulting mixture was heated at 80 °C for 2 hours. The reaction mixture was diluted with CH_2Cl_2 and washed with water. The combined organic layers were dried over anhydrous Na_2SO_4 and concentrated under reduced pressure to obtain the residue. The residue was purified over silica gel to afford **7a** (45 mg, 52%).

; ^{1}H NMR (400 MHz, $CDCl_3$) δ 8.65 (d, *J* = 8.6 Hz, 1H), 8.53 (ddd, *J* = 4.9, 1.8, 0.9 Hz, 2H), 7.87 (d, *J* = 8.7 Hz, 1H), 7.67 (d, *J* = 8.0 Hz, 1H), 7.64 (dd, *J* = 7.5, 1.8 Hz, 2H), 7.59 – 7.52 (m, 4H), 7.42 (d, *J* = 7.9 Hz, 1H), 7.14 (ddd, *J* = 7.4, 4.9, 1.3 Hz, 2H), 6.92 (d, *J* = 8.6 Hz, 2H), 4.02 (s, 2H), 3.90 (s, 4H), 3.85 (s, 3H), 1.57 (s, 9H); ^{13}C NMR (100 MHz, $CDCl_3$) δ 161.24, 160.51, 159.82, 152.26, 149.61, 147.88, 140.83, 136.98, 135.66, 133.70, 130.05, 129.37, 123.70, 122.60, 122.47, 121.03, 119.99, 115.53, 114.69, 95.21, 85.25, 84.09, 61.31, 60.98, 55.90, 28.28; HRMS–FAB: m/z $[M + H]^+$ calcd for $C_{36}H_{35}N_4O_4$: 587.2658; found:587.2662.

2-((bis(pyridin-2-ylmethyl)amino)methyl)-5-((4-cyanophenyl)ethynyl)quinolin-8-yl *tert*-butyl carbonate (7b)

Na_2CO_3 (14 mg, 0.13 mmol) was added to a well-stirred solution of **6b** (70 mg, 0.15 mmol) and bis(pyridin-2-ylmethyl)amine (24 mg, 0.12 mol) in CH_3CN (6 mL) at rt. The resulting mixture was heated at 80 °C for 2 hours. The reaction mixture was diluted with CH_2Cl_2 and washed with water. The combined organic layers were dried over anhydrous Na_2SO_4 and concentrated under reduced pressure to obtain the residue. The residue was purified over silica gel to afford **7b** (38 mg, 44%).

; ^{1}H NMR (400 MHz, $CDCl_3$) δ 8.58 (d, *J* = 8.7 Hz, 1H), 8.52 (ddd, *J* = 4.9, 1.8, 0.9 Hz, 2H), 7.90 (d, *J* = 8.7 Hz, 1H), 7.72 (dd, *J* = 7.9, 0.4 Hz, 1H), 7.67 (s, 4H), 7.66 – 7.59 (m, 3H), 7.55 (dd, *J* = 7.9, 1.1 Hz, 2H), 7.45 (d, *J* = 7.8 Hz, 1H), 7.13 (ddd, *J* = 7.4, 4.9, 1.3 Hz, 1H), 4.02 (s, 2H), 3.88 (s, 4H), 1.57 (s, 9H); ^{13}C NMR (100 MHz, $CDCl_3$) δ 151.65, 149.06, 148.40, 140.36, 136.75, 134.80, 132.27, 132.22, 130.71, 128.95, 127.86, 123.45, 122.54, 122.26, 120.66, 118.48, 117.99, 112.04, 92.80, 90.32, 84.50, 84.34, 60.78, 60.28, 27.81 (one carbon overlapped); HRMS–FAB: m/z $[M + H]^+$ calcd for $C_{36}H_{32}N_5O_3$: 582.2505; found:582.2508.

2-((bis(pyridin-2-ylmethyl)amino)methyl)-5-((4-methoxyphenyl)ethynyl)quinolin-8-ol (**2a**)
piperidine (0.015 mL, 0.15 mmol) was added to a well-stirred solution of **7a** (30 mg, 0.051 mmol) in CH_2Cl_2 (2 mL) at rt for 10 min. The residue was concentrated under reduced pressure to obtain the residue. The residue was purified over silica gel to afford **2a** (10 mg, 43%).
^{1}H NMR (400 MHz, $CDCl_3$) δ 8.63 – 8.54 (m, 3H), 7.71 – 7.62 (m, 4H), 7.59 – 7.49 (m, 4H), 7.16 (ddd, *J* = 7.4, 4.9, 1.2 Hz, 2H), 7.11 (d, *J* = 8.0 Hz, 1H), 6.90 (d, *J* = 8.9 Hz, 2H), 4.04 (s, 2H), 3.95 (s, 4H), 3.84 (s, 3H); ^{13}C NMR (100 MHz, $CDCl_3$) δ 159.68, 159.26, 157.60, 153.09, 149.29, 137.43, 136.54, 135.39, 133.02, 131.61, 127.87, 123.32, 122.37, 122.25, 115.68, 114.17, 111.33, 110.20, 92.52, 85.36, 60.26, 59.41, 55.43; HRMS–EI: m/z $[M]^+$ calcd for $C_{31}H_{26}N_4O_2$: 486.2056; found:482.2059.

4-((2-((bis(pyridin-2-ylmethyl)amino)methyl)-8-hydroxyquinolin-5-yl)ethynyl)benzonitrile (**2b**)
piperidine (0.015 mL, 0.15 mmol) was added to a well-stirred solution of **7b** (30 mg, 0.051 mmol) in CH_2Cl_2 (2 mL) at rt for 10 min. The residue was concentrated under reduced pressure to obtain the residue. The residue was purified over silica gel to afford **2b** (10 mg, 40%).
^{1}H NMR (400 MHz, $CDCl_3$) δ 8.59 (ddd, *J* = 4.9, 1.8, 0.9 Hz, 2H), 8.54 (d, *J* = 8.6 Hz, 1H), 7.72 – 7.68 (m, 2H), 7.68 – 7.64 (m, 6H), 7.58 – 7.52 (m, 2H), 7.20 – 7.13 (m, 3H), 4.08 (s, 2H), 3.97 (s, 4H); ^{13}C NMR (100 MHz, $CDCl_3$) δ 154.51, 149.28, 137.49, 137.43, 136.83, 135.09, 132.96, 132.20, 131.93, 128.52, 127.96, 123.74, 122.98, 122.59, 122.50, 118.65, 111.34, 110.71, 109.67, 91.51, 91.16, 59.93, 58.86; HRMS–EI: m/z $[M]^+$ calcd for $C_{31}H_{23}N_5O$: 481.1903; found:481.1906.

Binding Studies
Binding constants by fluorescence titrations

Upon addition of incremental amounts of Cd^{2+} to the solution of **2b** (from 0 equiv. to 1.6 equiv. of Cd^{2+}), fluorescence change of **2b** (10 μM) were recorded in PBS buffer (10 mM, pH 7.2) containing 50% MeOH. Equilibrium constants of complexes were calculated using the Hill equation: $\log(Y/(1 - Y)) = n \log[Cd^{2+}] + \log K$, where Y is the fractional saturation of the host, n is the Hill coefficient, and K is the association constant.

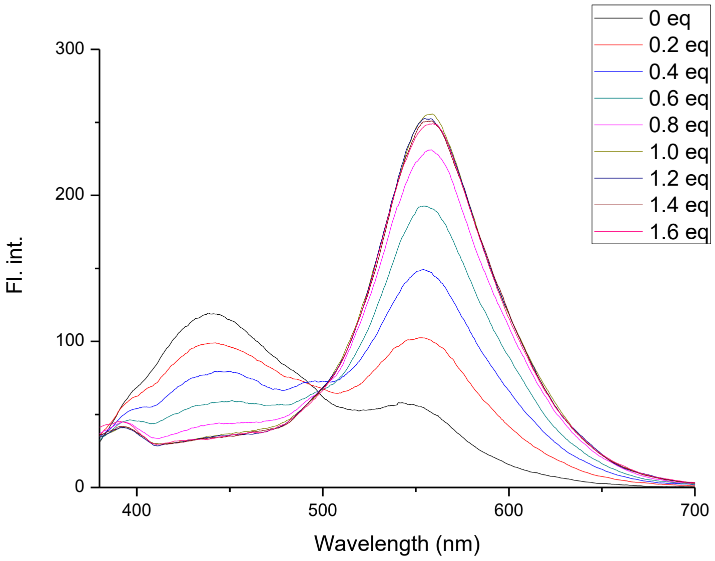

Figure S1. Fluorescence titration of **2b** (10 μM) recorded with various amount of Cd^{2+} (from 0 to 1.6 equiv.)

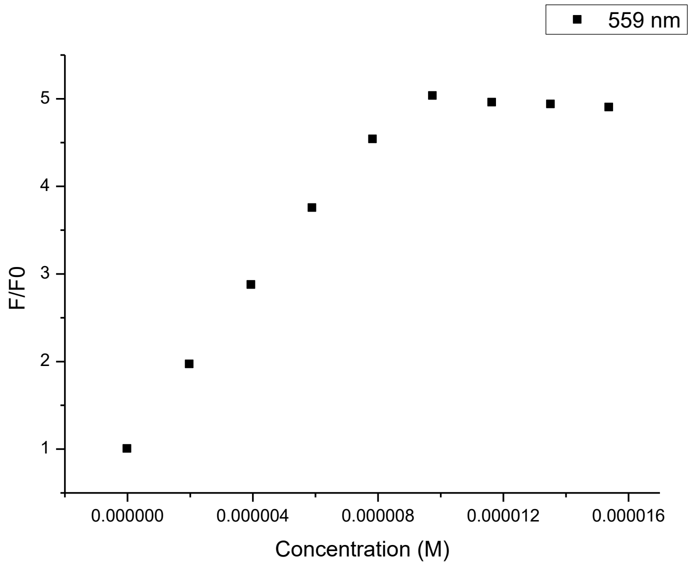

Figure S2. Fluorescence titration curve of **2b** (10 μM) obtained at 559 nm as a function of Cd^{2+} concentration.

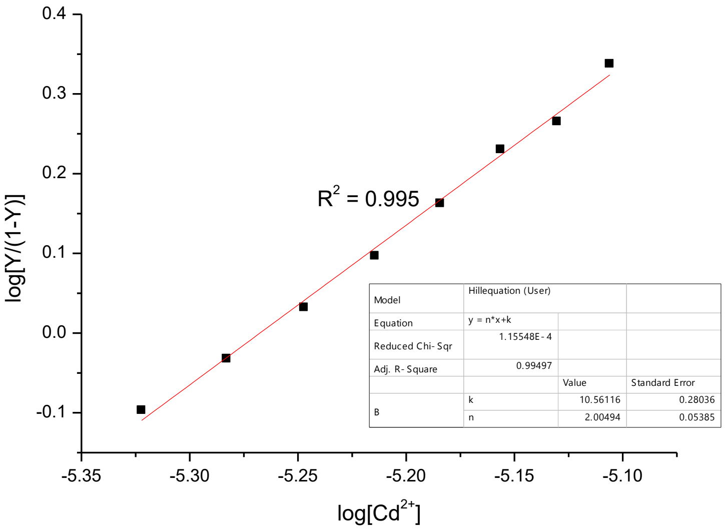

Figure S3. Hill plot of **2b** obtained at 559 nm as a function of Cd^{2+} concentration

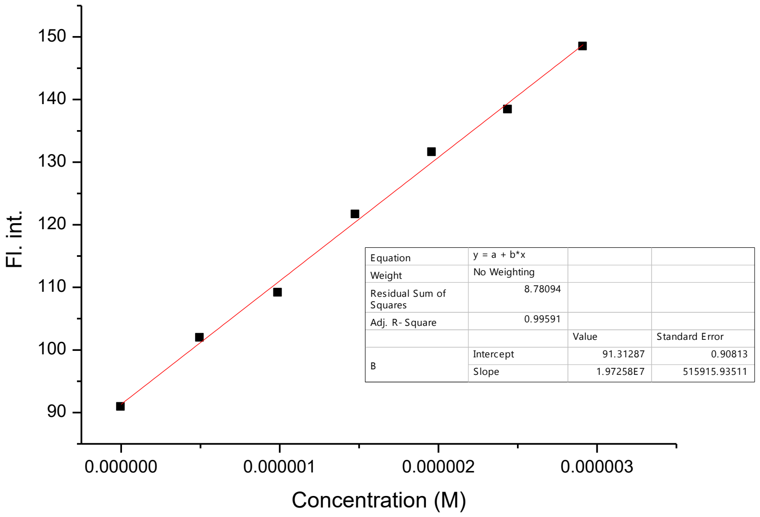

Figure S4. Linear plot of **2b** (10 μM) obtained at 559 nm as a function of Cd^{2+} concentration

Binding constants by UV-vis titrations

Upon addition of incremental amounts of Cd^{2+} to the solution of **2b** (from 0 equiv. to 1.8 equiv. of Cd^{2+}), absorbance change of **2b** (10 μM) were recorded in PBS buffer (10 mM, pH 7.2) containing 50% MeOH. Equilibrium constants of complexes were calculated using the Hill equation: $\log(Y/(1 - Y)) = n \log[Ag^+] + \log K$, where Y is the fractional saturation of the host, n is the Hill coefficient, and K is the association constant.

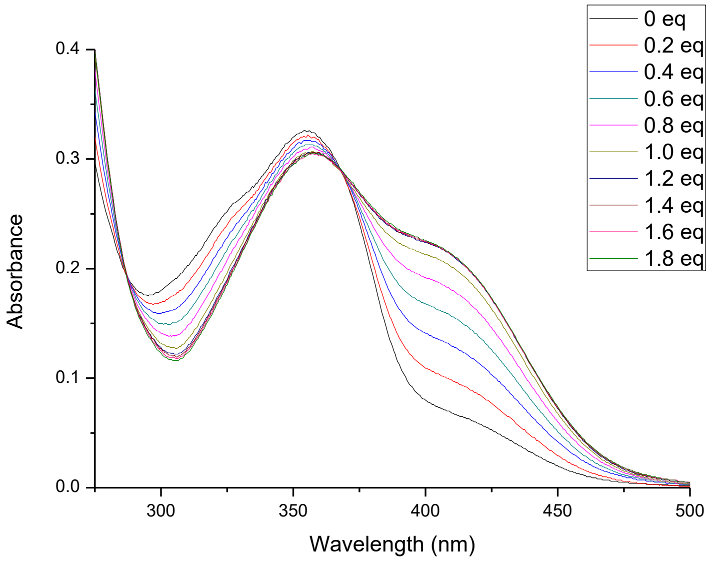

Figure S5. UV-vis titration of **2b** (20 μM) recorded with various amount of Cd^{2+} (from 0 to 1.8 equiv.)

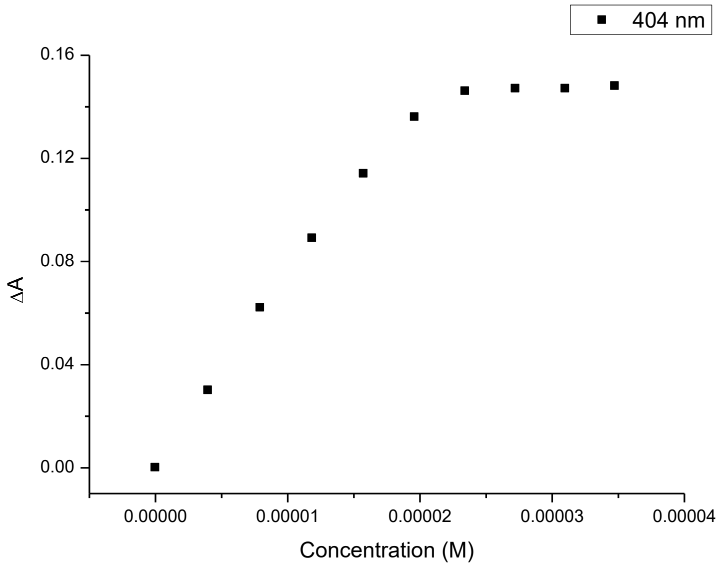

Figure S6. UV-vis titration curve of **2b** (10 μM) obtained at 404 nm as a function of Cd^{2+} concentration

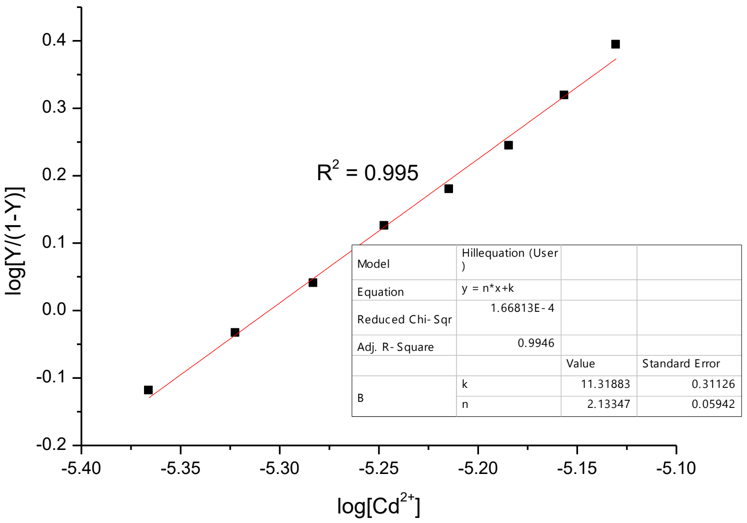

Figure S7. Hill plot of **2b** (10 μM) obtained at 404 nm as a function of Cd^{2+} concentration

DFT Calculations

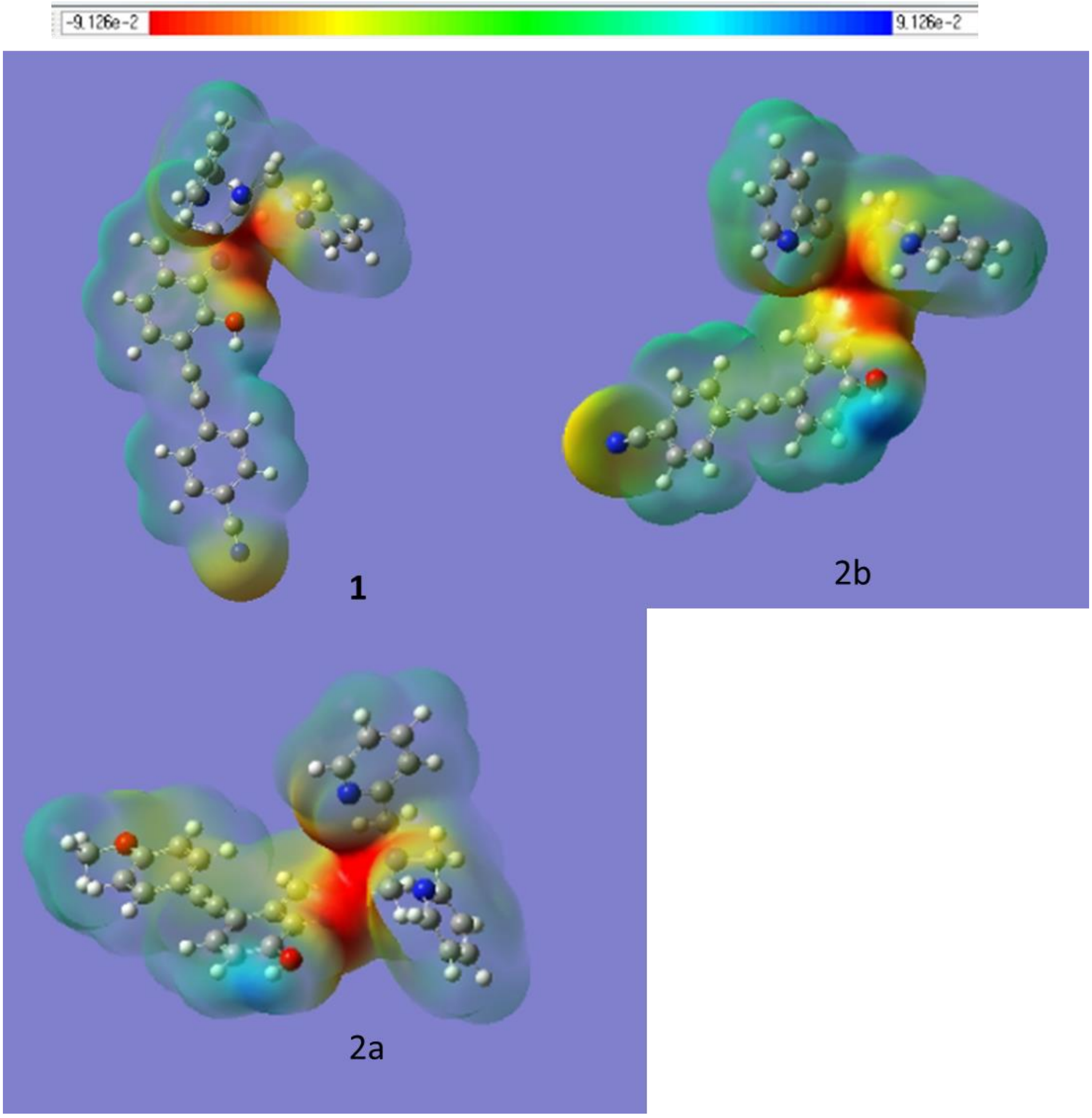

Figure S8. Electrostatic potential maps of tolans (**1**, **2a**, and **2b)** obtained by DFT calculations (B3LYP/6-31G**)

Table S1. The energy levels of optimized DFT calculation of metal complexes of **2b** and **1** based on density functional theory (B3LYP/6-31G**)

	Hartrees	Kcal/mol
[(**1**-H)AlHCO_2]$^+$	-1975.154785	-1239409.627
[(**2b**-H)AlHCO_2]$^+$	-1975.155214	-1239409.897
[(**1**-H)Cd]$^+$	-1591.426433	-998620.0869
[(**2b**-H)Cd]$^+$	-1591.427564	-998620.7962

Table S2. DFT calculation of Cd^{2+} complex of **2b** ([Cd(**2b**-H)]$^{+}$) based on density functional theory (B3LYP/6-31G**)

Row	Highlight	Display	Tag	Symbol	X	Y	Z
1	No	Show	1	C	1.147826	-0.55548	-1.10191
2	No	Show	2	C	0.049056	-1.85735	0.450348
3	No	Show	3	C	-1.2244	-1.29234	0.135191
4	No	Show	4	C	-1.24537	-0.31319	-0.88496
5	No	Show	5	C	-0.07206	0.050943	-1.5022
6	No	Show	6	C	0.123191	-2.86972	1.46642
7	No	Show	7	C	-2.4049	-1.72719	0.833811
8	No	Show	8	H	-2.19239	0.135678	-1.16648
9	No	Show	9	H	-0.06961	0.794748	-2.29323
10	No	Show	10	C	-2.27343	-2.70508	1.814151
11	No	Show	11	C	-1.02638	-3.27037	2.126561
12	No	Show	12	H	-3.15639	-3.03893	2.348648
13	No	Show	13	C	2.463518	-0.17249	-1.75434
14	No	Show	14	H	3.034749	-1.09407	-1.88565
15	No	Show	15	H	2.267827	0.246097	-2.76271
16	No	Show	16	N	1.198424	-1.48689	-0.16574
17	No	Show	17	N	3.273352	0.737451	-0.93693
18	No	Show	18	C	4.667581	0.780183	-1.39258
19	No	Show	19	H	5.166524	1.583067	-0.84419
20	No	Show	20	H	4.738847	1.021521	-2.47243
21	No	Show	21	C	2.684134	2.078831	-0.8627
22	No	Show	22	H	1.616199	1.959411	-0.66364
23	No	Show	23	H	2.784297	2.624312	-1.82281
24	No	Show	24	C	3.2519	2.920951	0.263072
25	No	Show	25	C	4.134887	3.978302	0.014488
26	No	Show	26	C	3.314569	3.326474	2.52147
27	No	Show	27	C	4.621848	4.724494	1.087454
28	No	Show	28	H	4.431199	4.210734	-1.00424
29	No	Show	29	C	4.207649	4.390662	2.373706
30	No	Show	30	H	2.958818	3.044133	3.511115
31	No	Show	31	H	5.30952	5.548492	0.919799
32	No	Show	32	H	4.557207	4.939878	3.24212
33	No	Show	33	C	5.431656	-0.49783	-1.10708
34	No	Show	34	C	5.753386	-1.40169	-2.12658

35	No	Show	35	C	6.473746	-1.81081	0.460773
36	No	Show	36	C	6.462561	-2.56012	-1.81026
37	No	Show	37	H	5.453065	-1.19572	-3.14983
38	No	Show	38	C	6.829513	-2.77554	-0.48502
39	No	Show	39	H	6.753969	-1.93914	1.505175
40	No	Show	40	H	6.722002	-3.27717	-2.58392
41	No	Show	41	H	7.382243	-3.66071	-0.18668
42	No	Show	42	N	2.839388	2.606514	1.502301
43	No	Show	43	N	5.797576	-0.69684	0.17007
44	No	Show	44	C	-3.67834	-1.17503	0.536947
45	No	Show	45	C	-4.77555	-0.70643	0.289098
46	No	Show	46	C	-6.06262	-0.17214	0.01641
47	No	Show	47	C	-7.19445	-0.63036	0.725534
48	No	Show	48	C	-6.23847	0.826092	-0.96625
49	No	Show	49	C	-8.4532	-0.1112	0.462803
50	No	Show	50	H	-7.06921	-1.39682	1.482454
51	No	Show	51	C	-7.49638	1.346615	-1.23069
52	No	Show	52	H	-5.3758	1.185806	-1.51633
53	No	Show	53	C	-8.61688	0.882923	-0.51852
54	No	Show	54	H	-9.31785	-0.46835	1.011813
55	No	Show	55	H	-7.62329	2.113586	-1.98713
56	No	Show	56	C	-9.91632	1.419553	-0.79105
57	No	Show	57	N	-10.9724	1.855646	-1.01291
58	No	Show	58	H	-0.96478	-4.03391	2.89848
59	No	Show	59	O	1.343327	-3.38822	1.727333
60	No	Show	60	H	1.262152	-4.03815	2.43886

NMR Studies

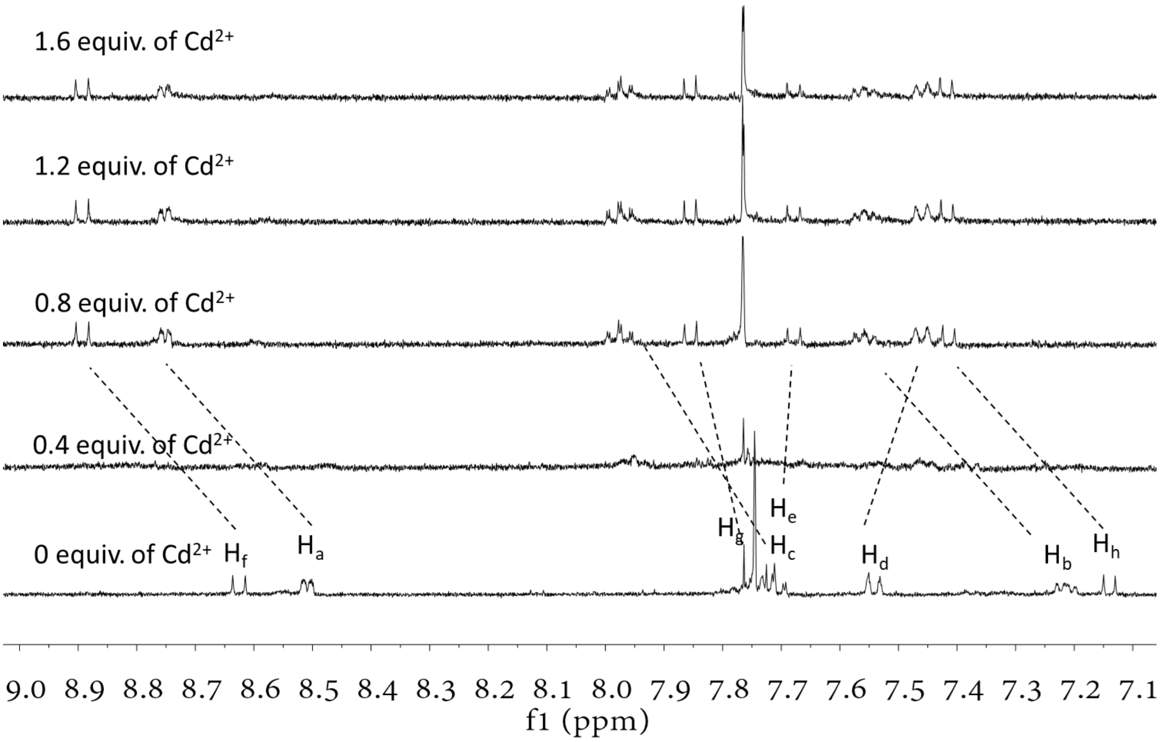

Figure S9. Partial ^{1}H-NMR spectra recorded during the titration of **2b (**[H] = 2.0 × 10^{-3} M in CD_3CN) with $Cd(ClO_4)_2$.

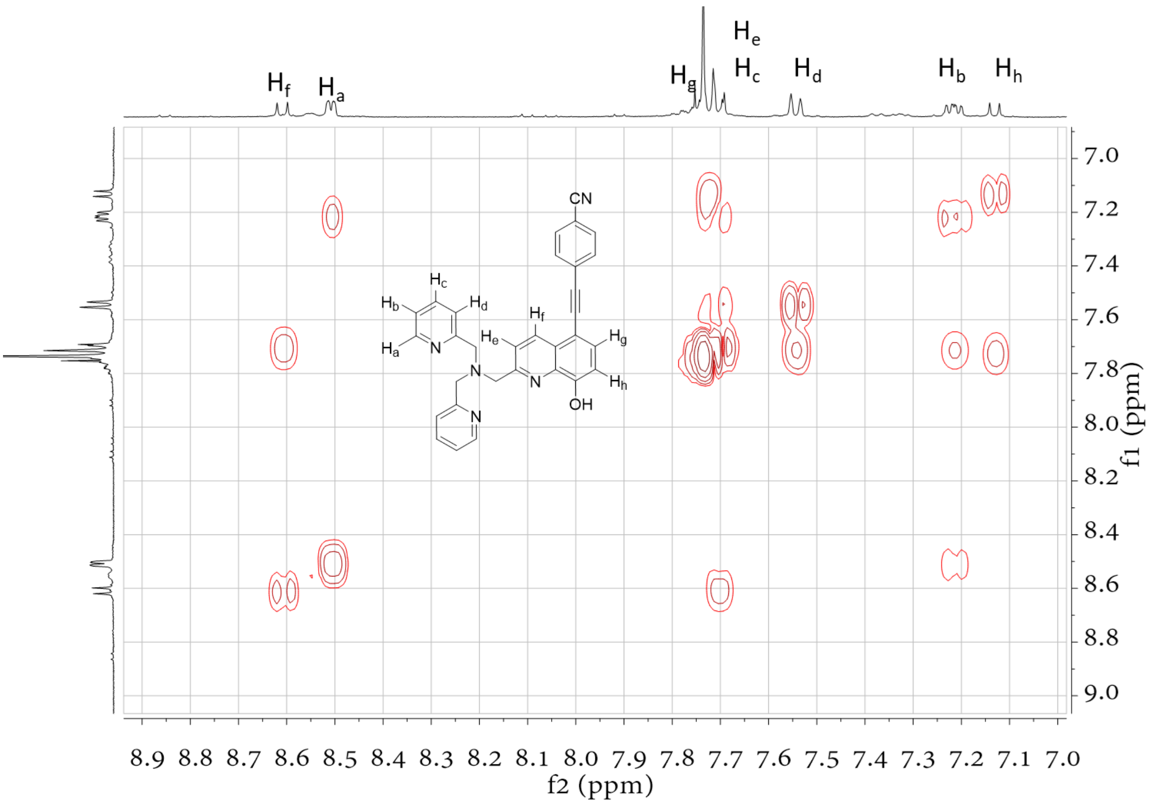

Figure S10. 2D COSY spectrum of **2b** in CD_3CN at 25 °C (400 MHz)

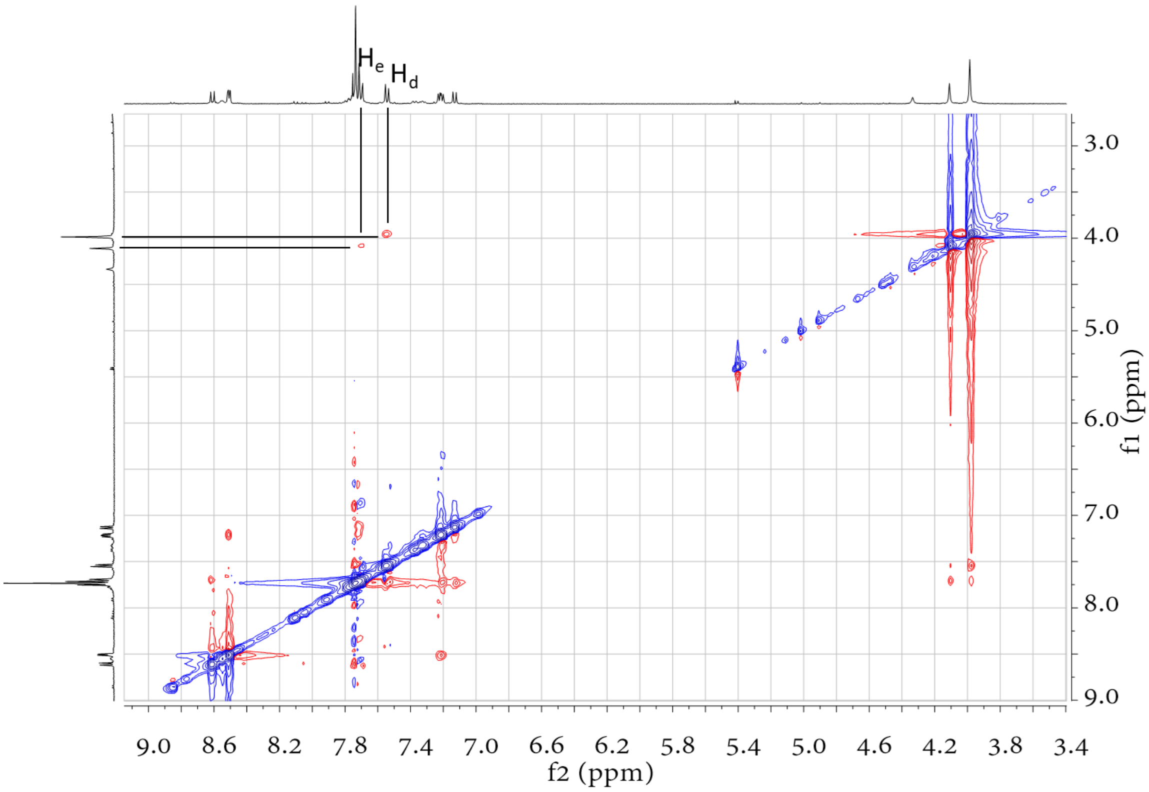

Figure S11. 2D NOESY spectrum of **2b** in CD_3CN at 25 °C (400 MHz)

NMR Spectra

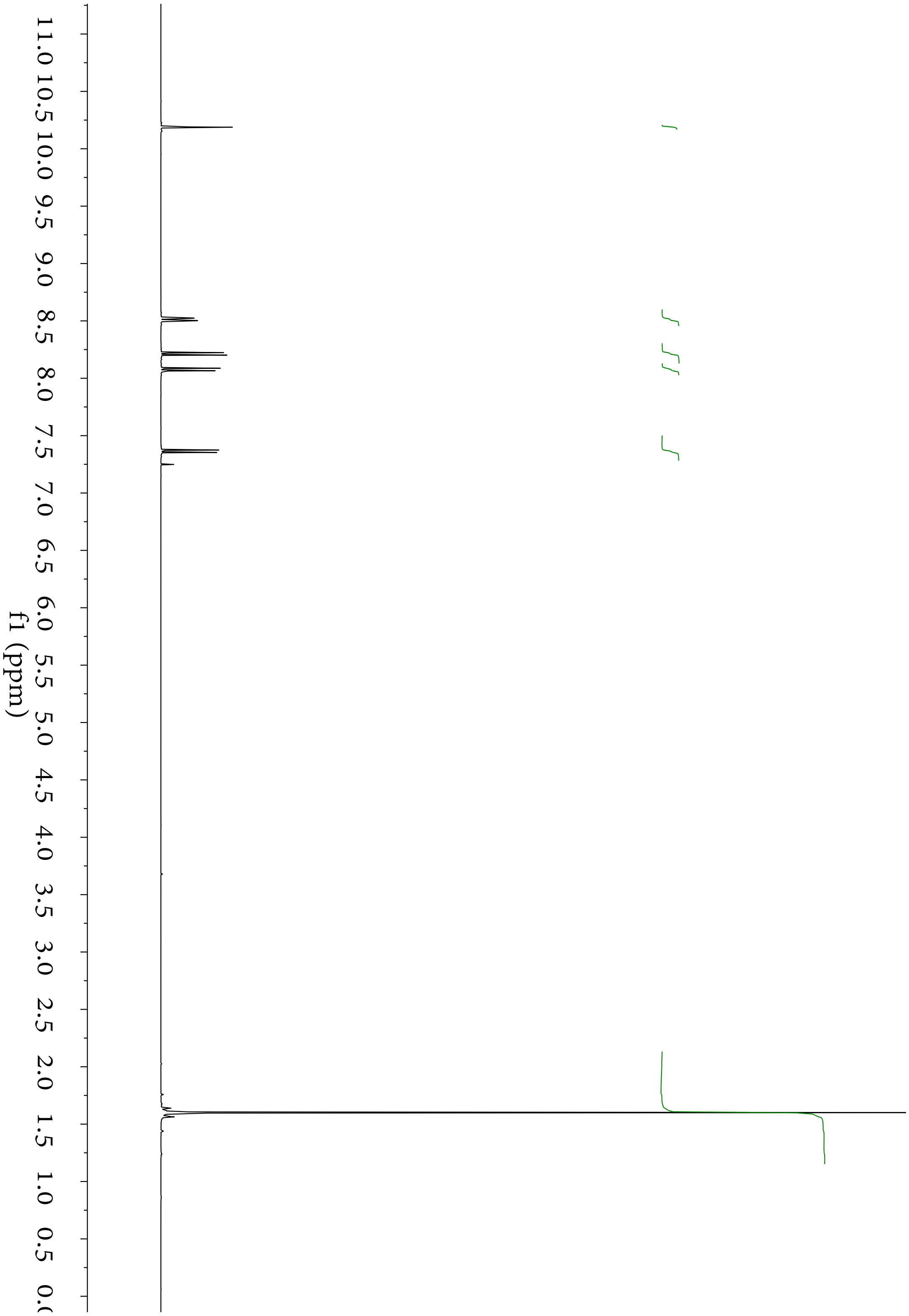

Figure S12. ^{1}H NMR spectrum of **4** recorded in $CDCl_3$

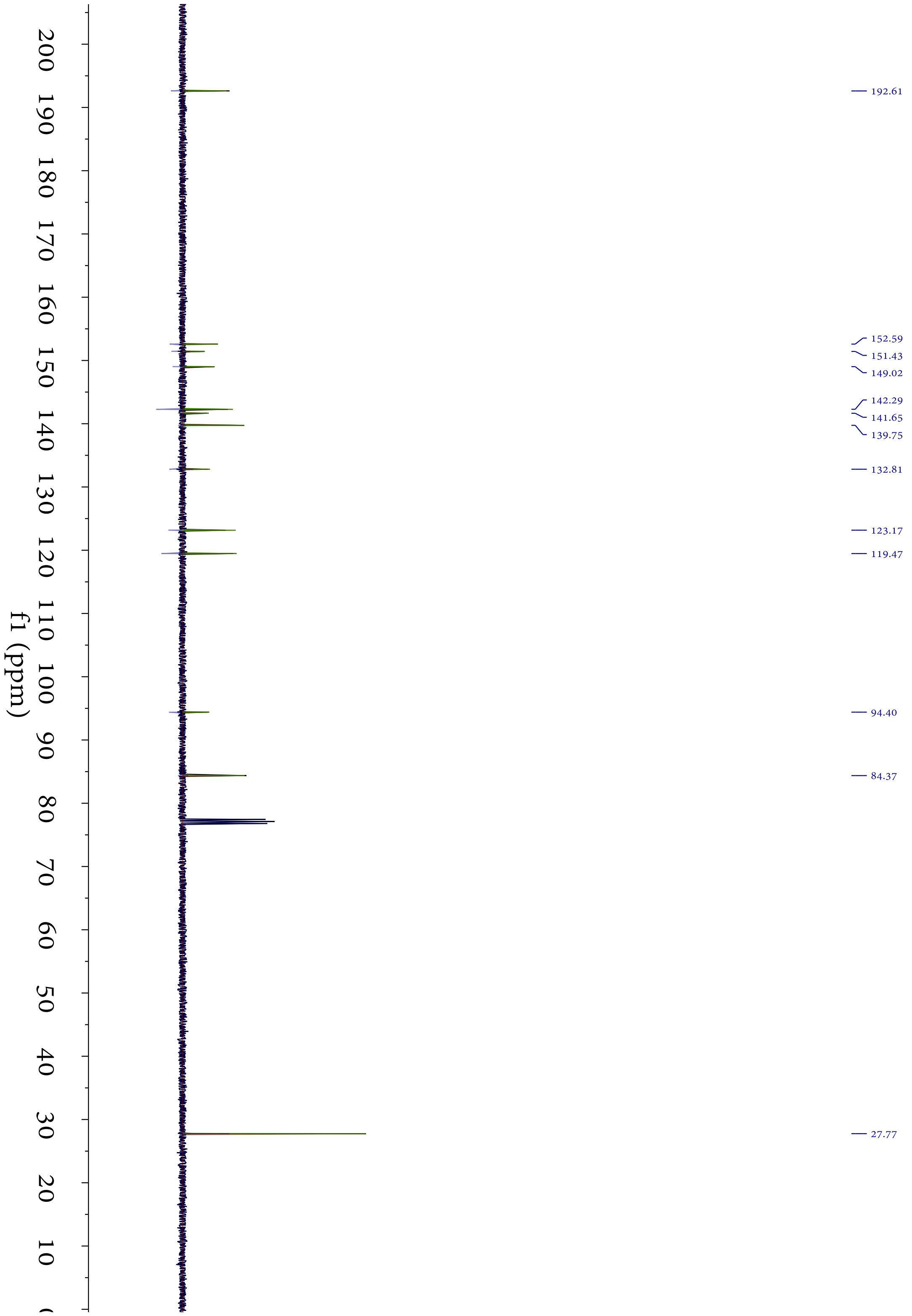

Figure S13. ^{13}C NMR spectrum of **4** recorded in $CDCl_3$

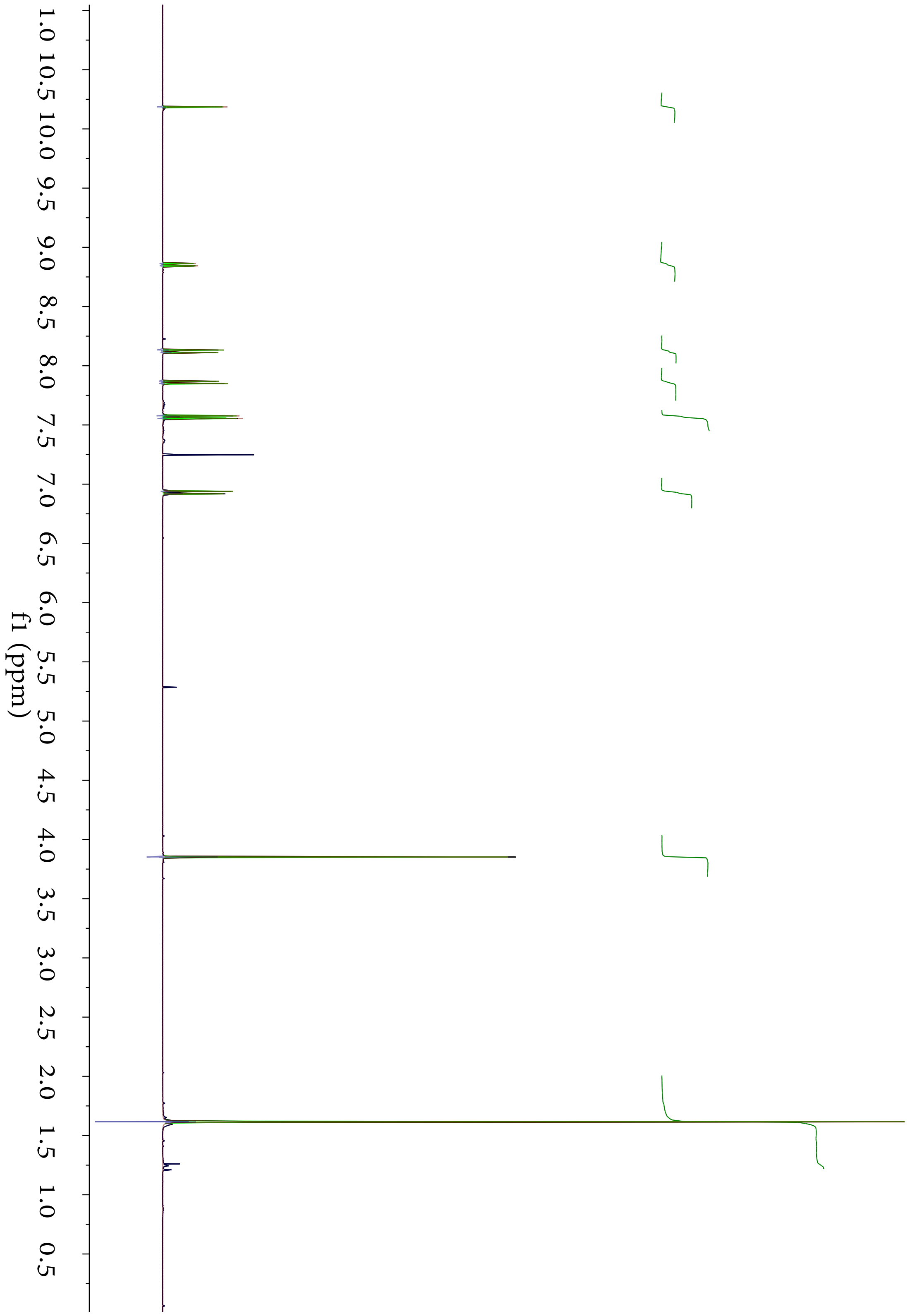

Figure S14. ^{1}H NMR spectrum of **5a** recorded in $CDCl_3$

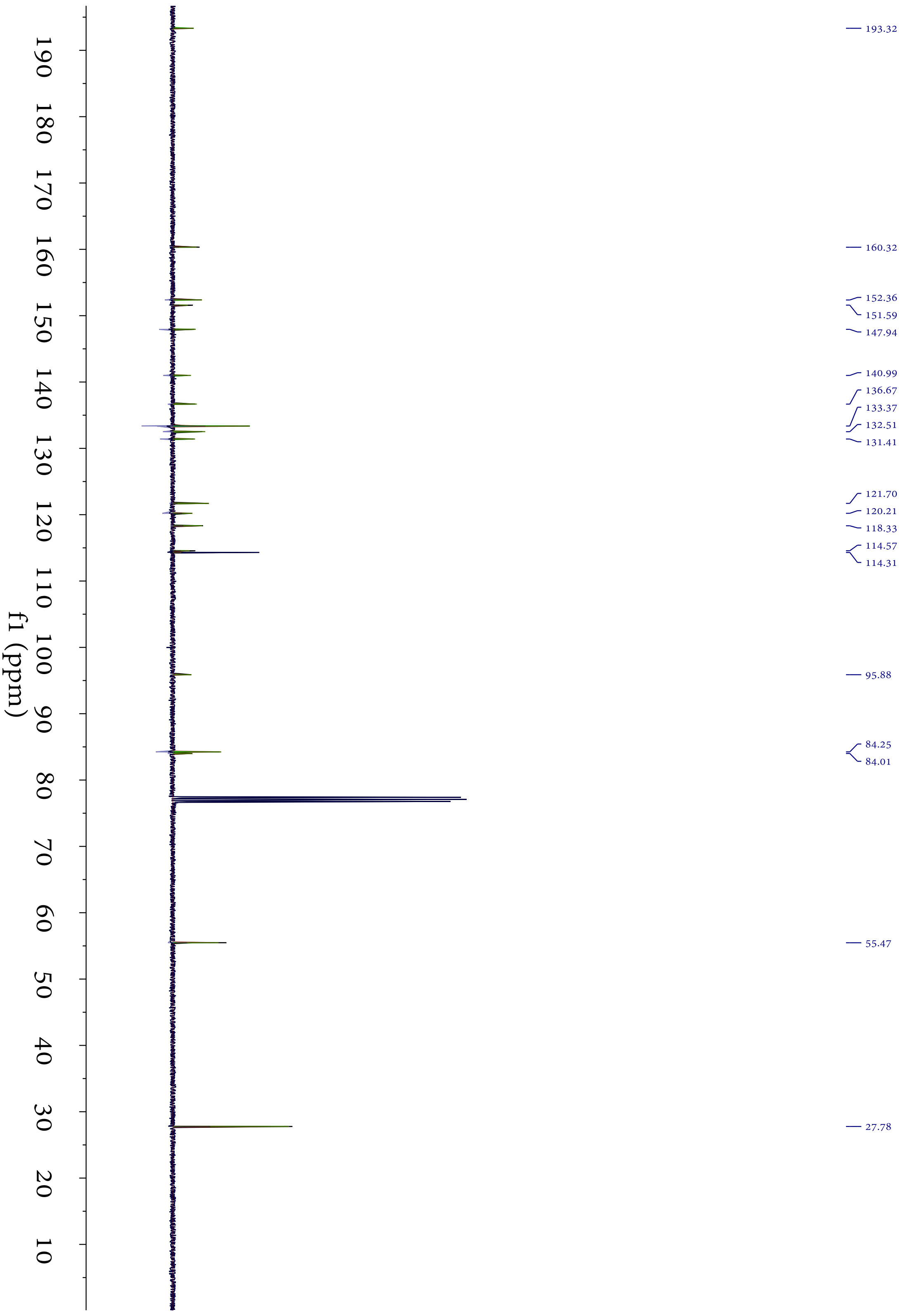

Figure S15. ^{13}C NMR spectrum of **5a** recorded in $CDCl_3$

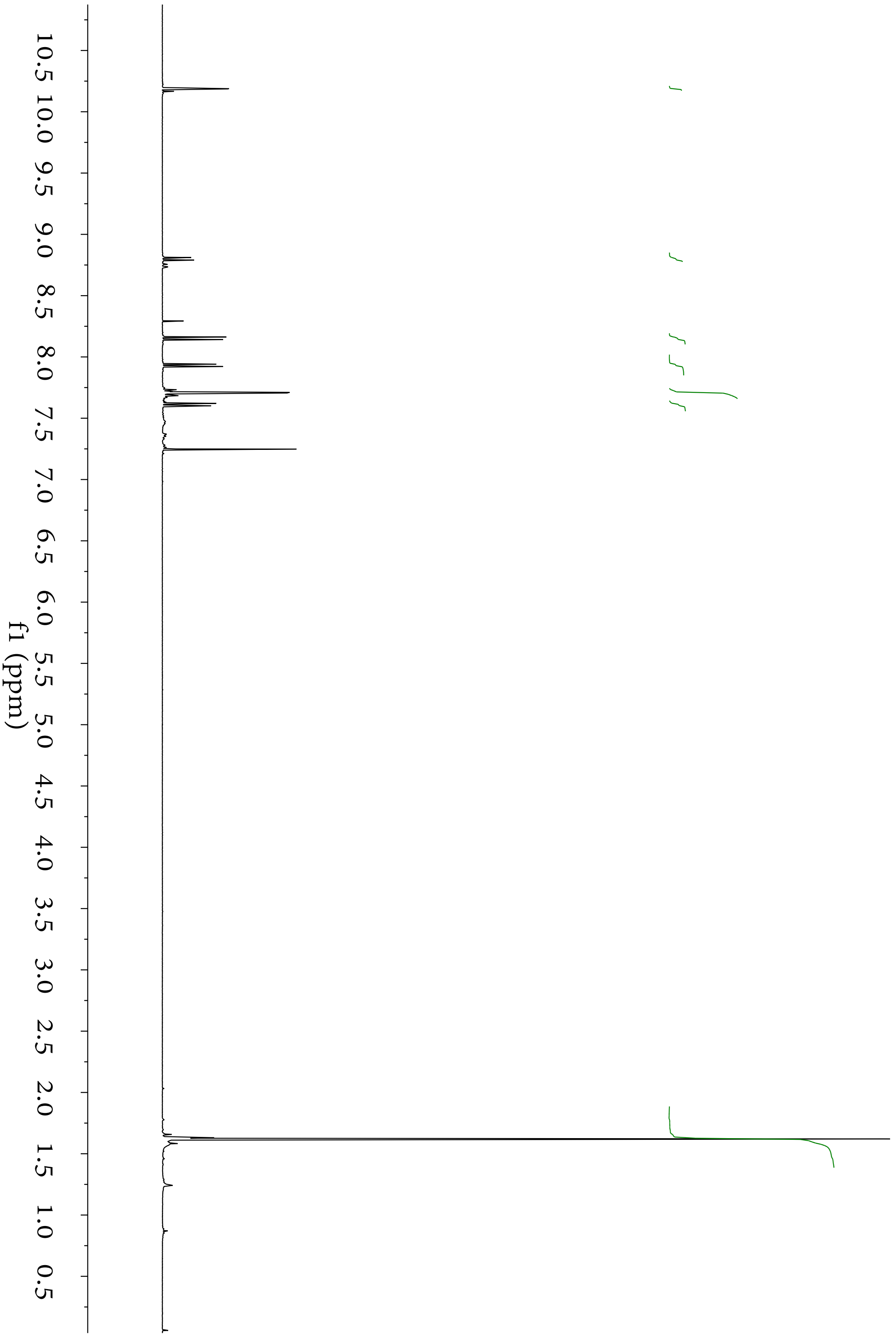

Figure S16. ^{1}H NMR spectrum of **5b** recorded in $CDCl_3$

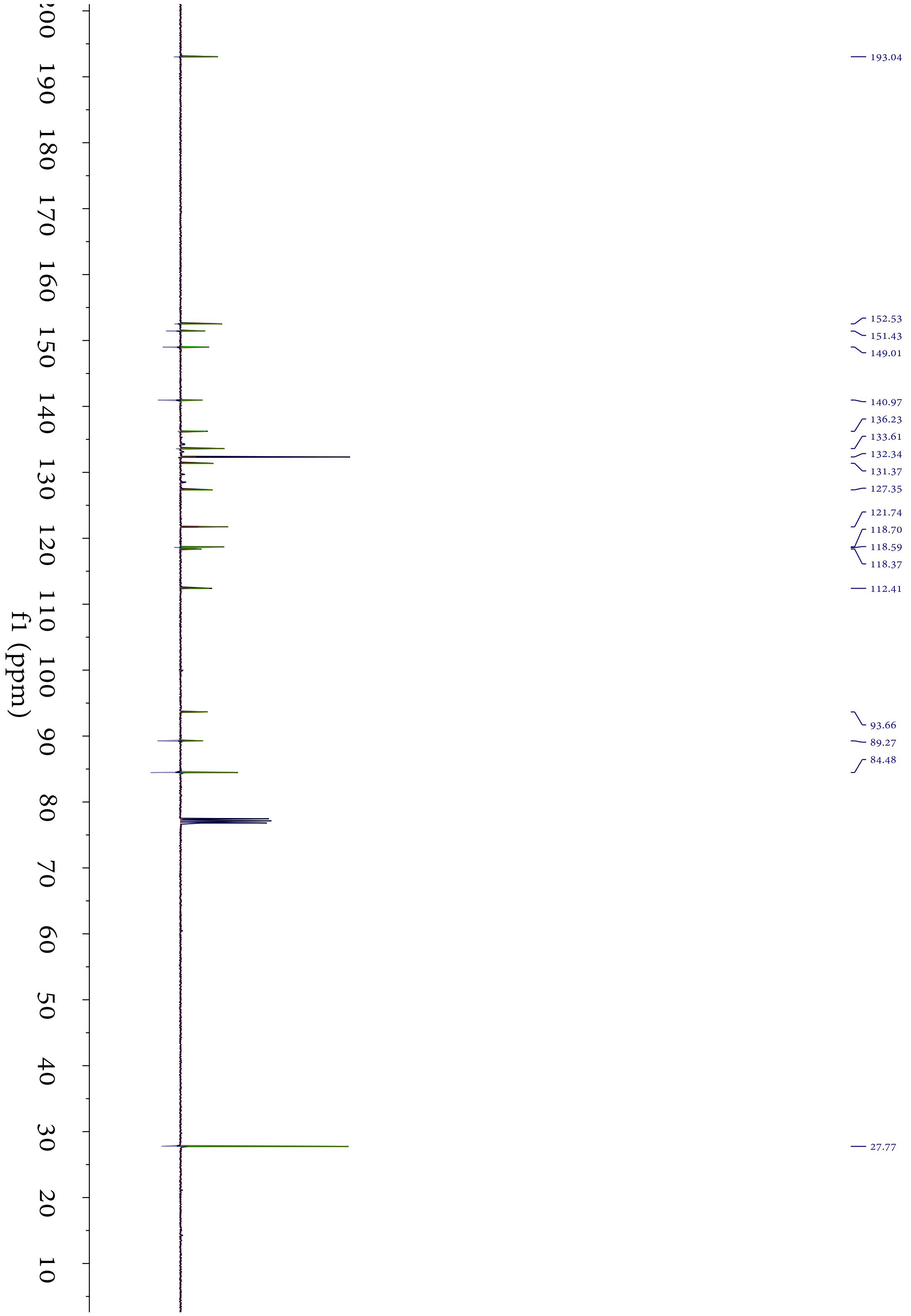

Figure S17. ^{13}C NMR spectrum of **5b** recorded in $CDCl_3$

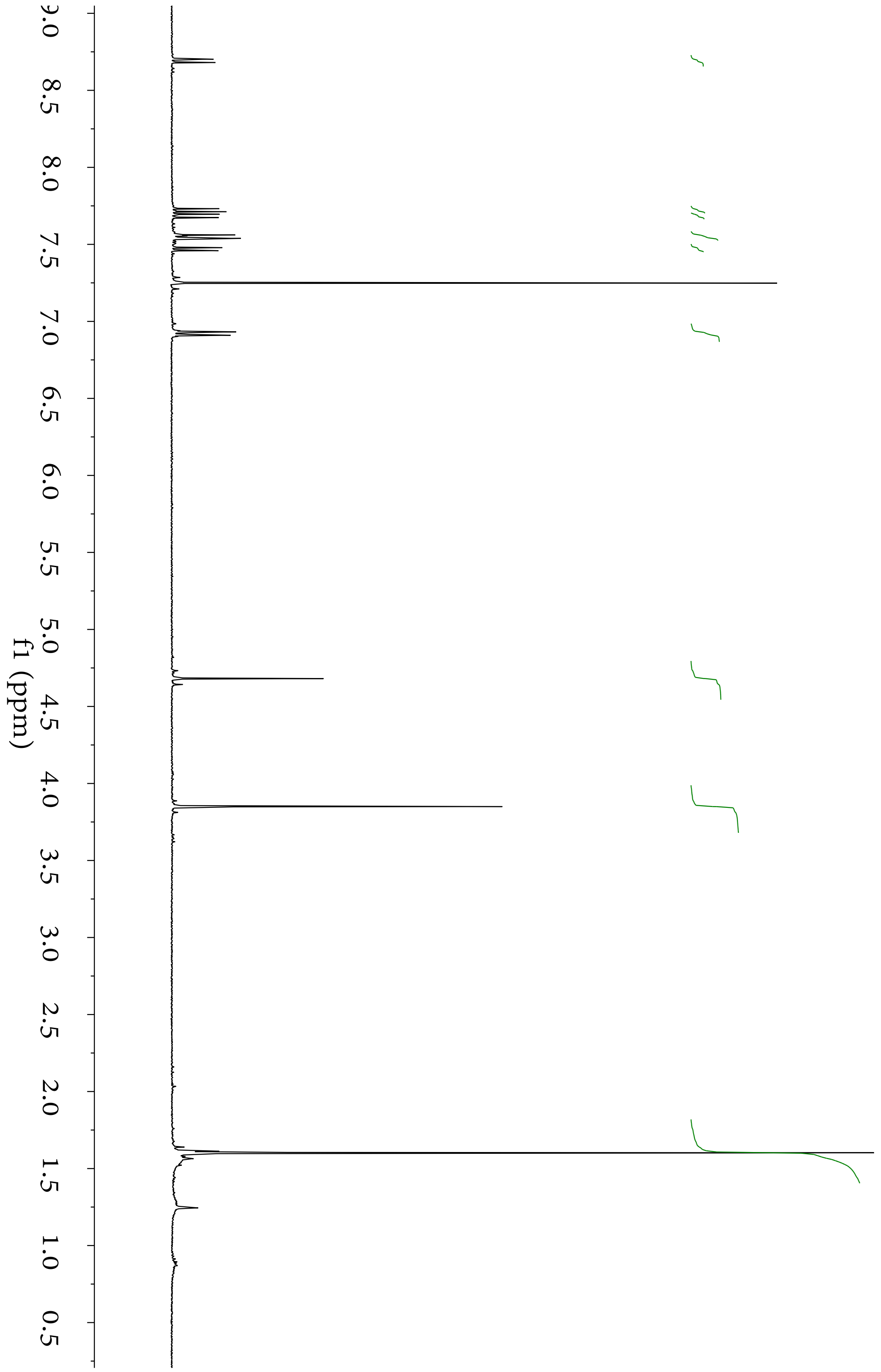

Figure S18. ^{1}H NMR spectrum of **6a** recorded in $CDCl_3$

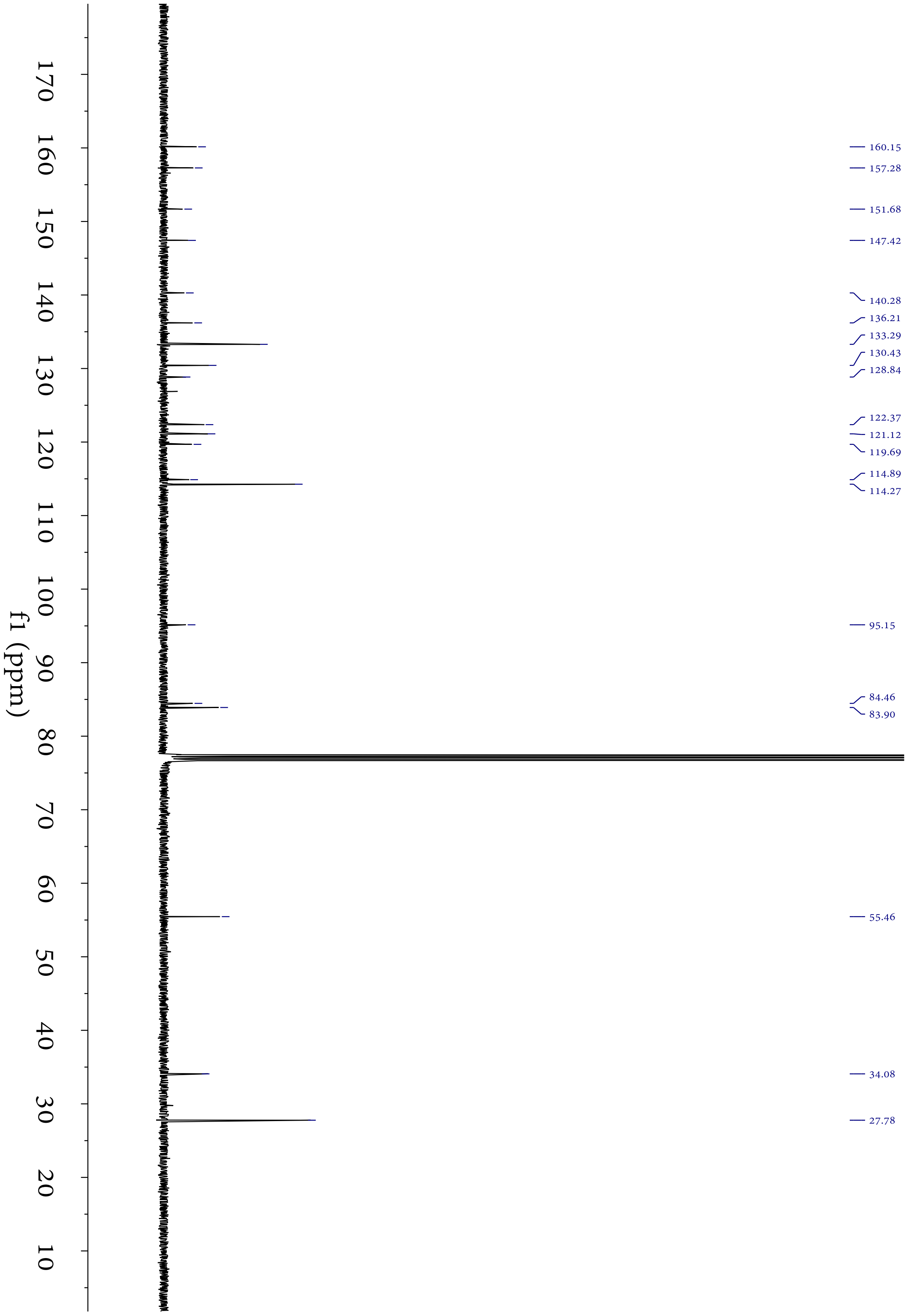

Figure S19. ^{13}C NMR spectrum of **6a** recorded in $CDCl_3$

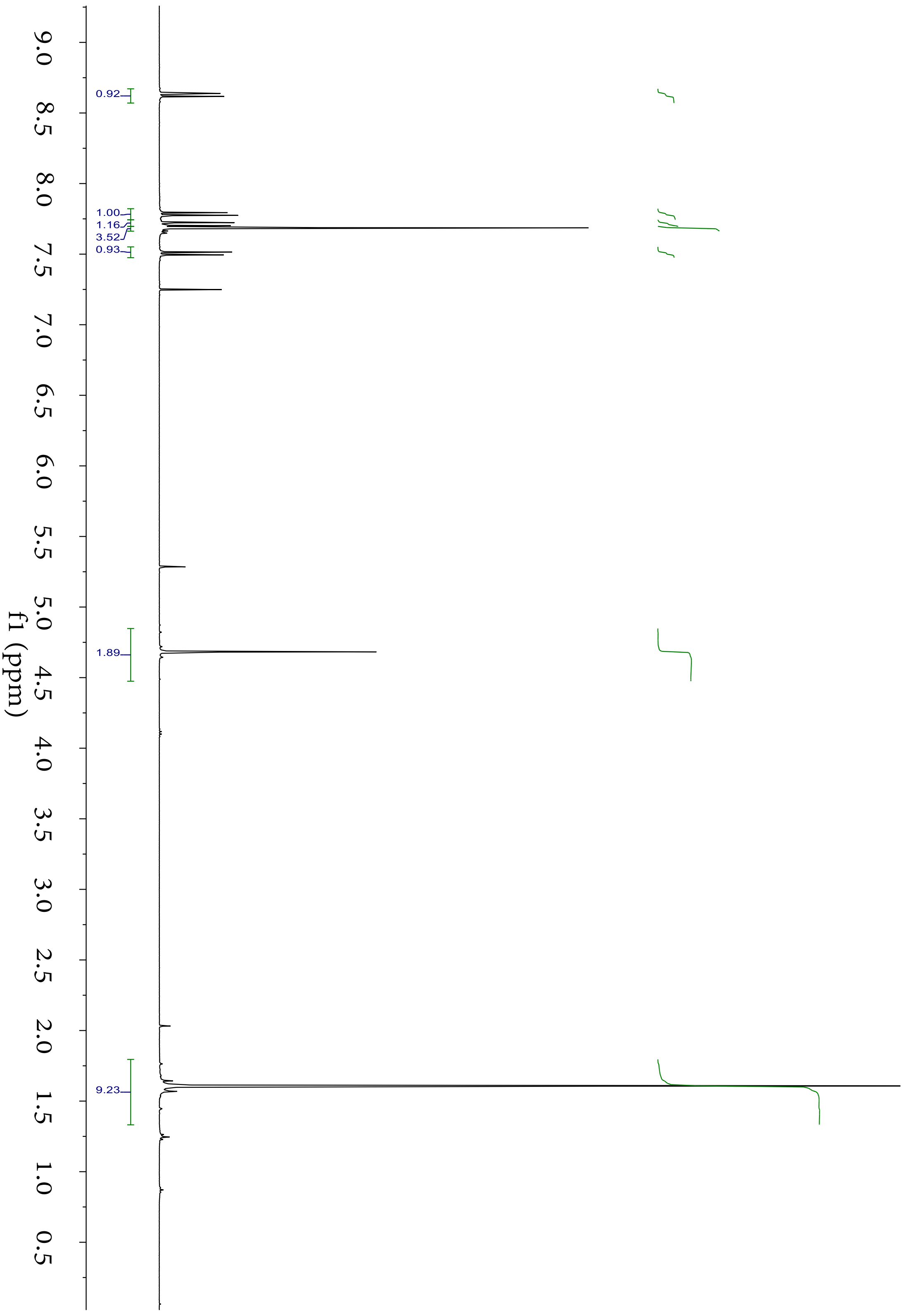

Figure S20. ^{1}H NMR spectrum of **6b** recorded in $CDCl_3$

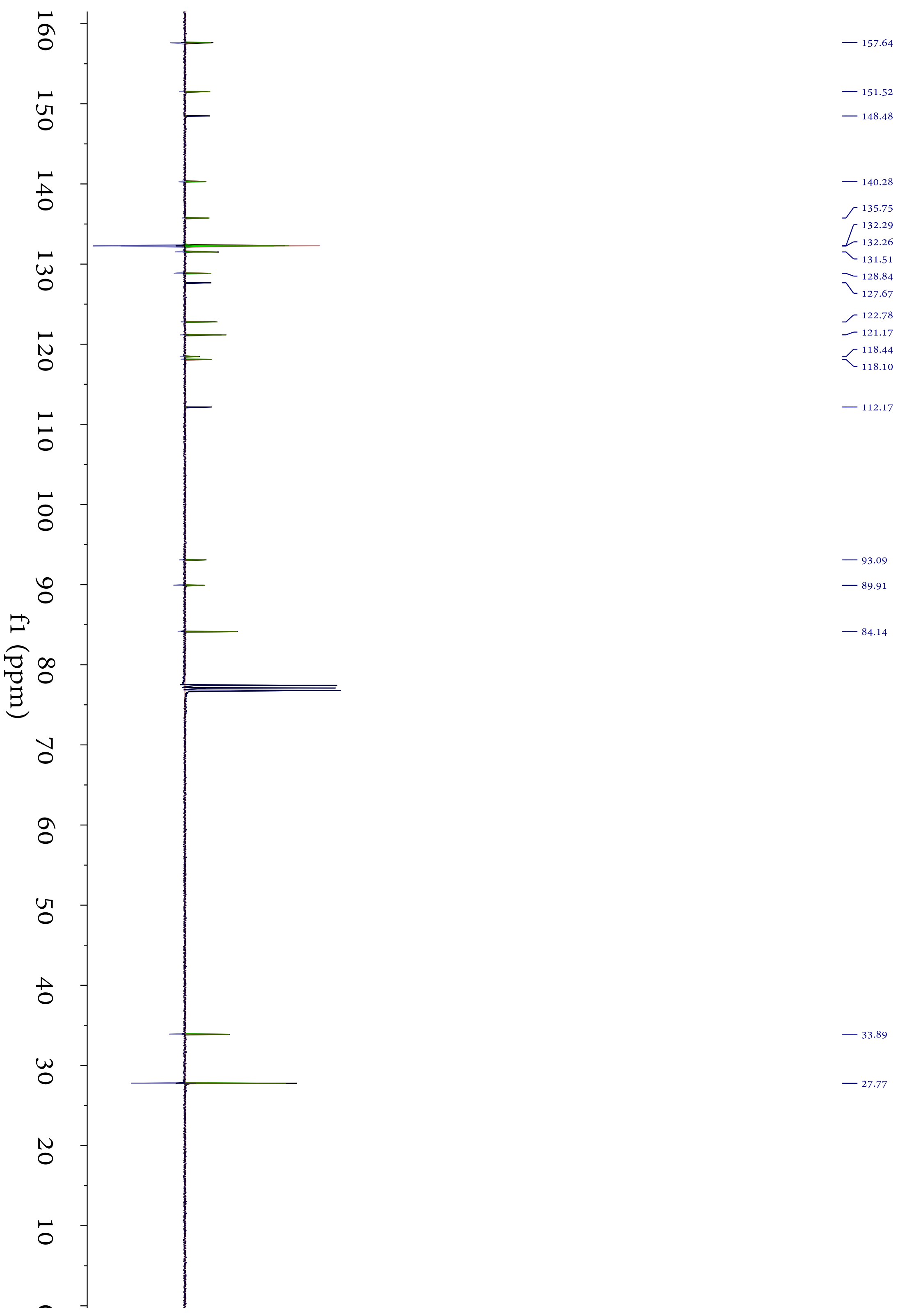

Figure S21. ^{13}C NMR spectrum of **6b** recorded in $CDCl_3$

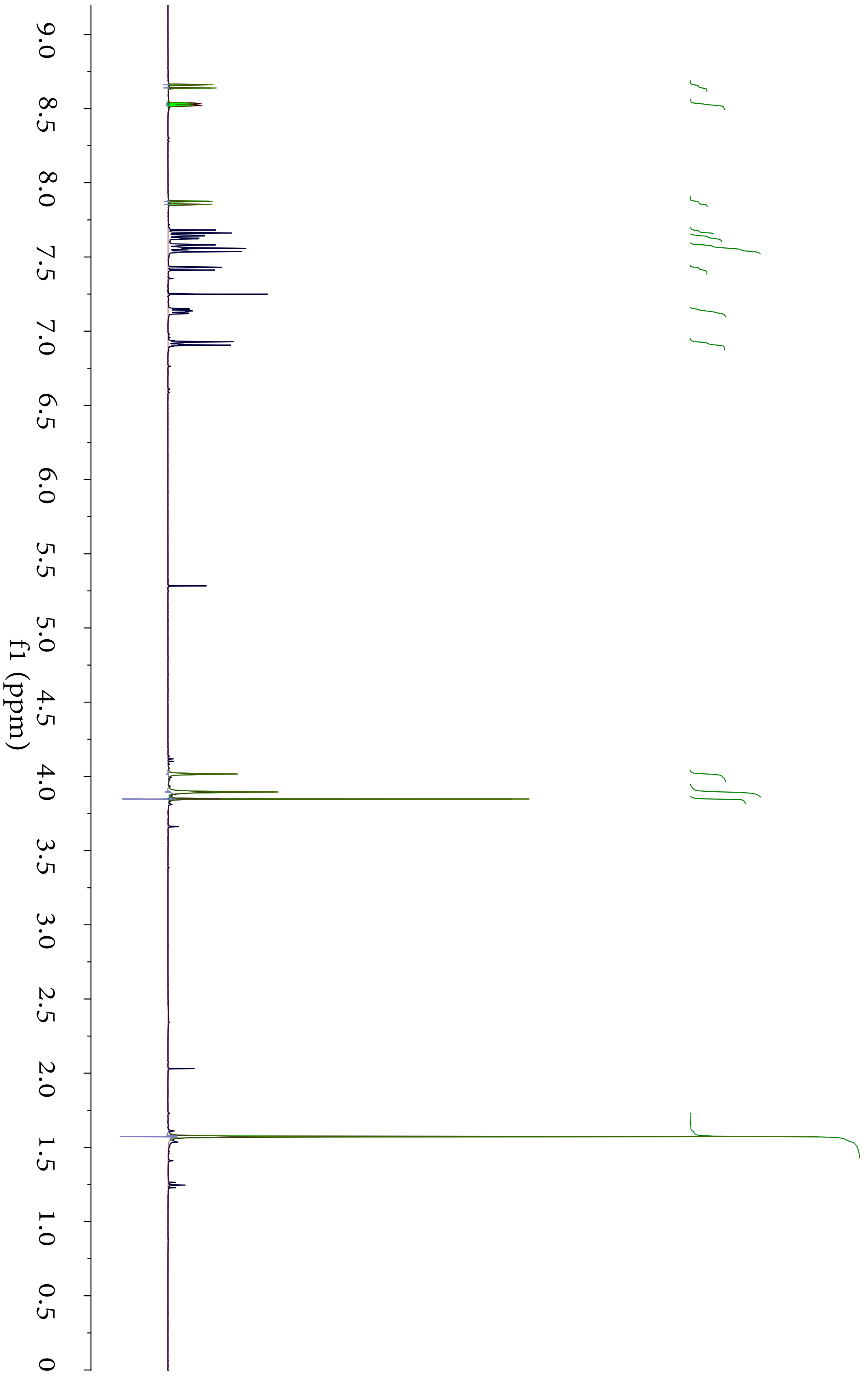

Figure S22. ^{1}H NMR spectrum of **7a** recorded in $CDCl_3$

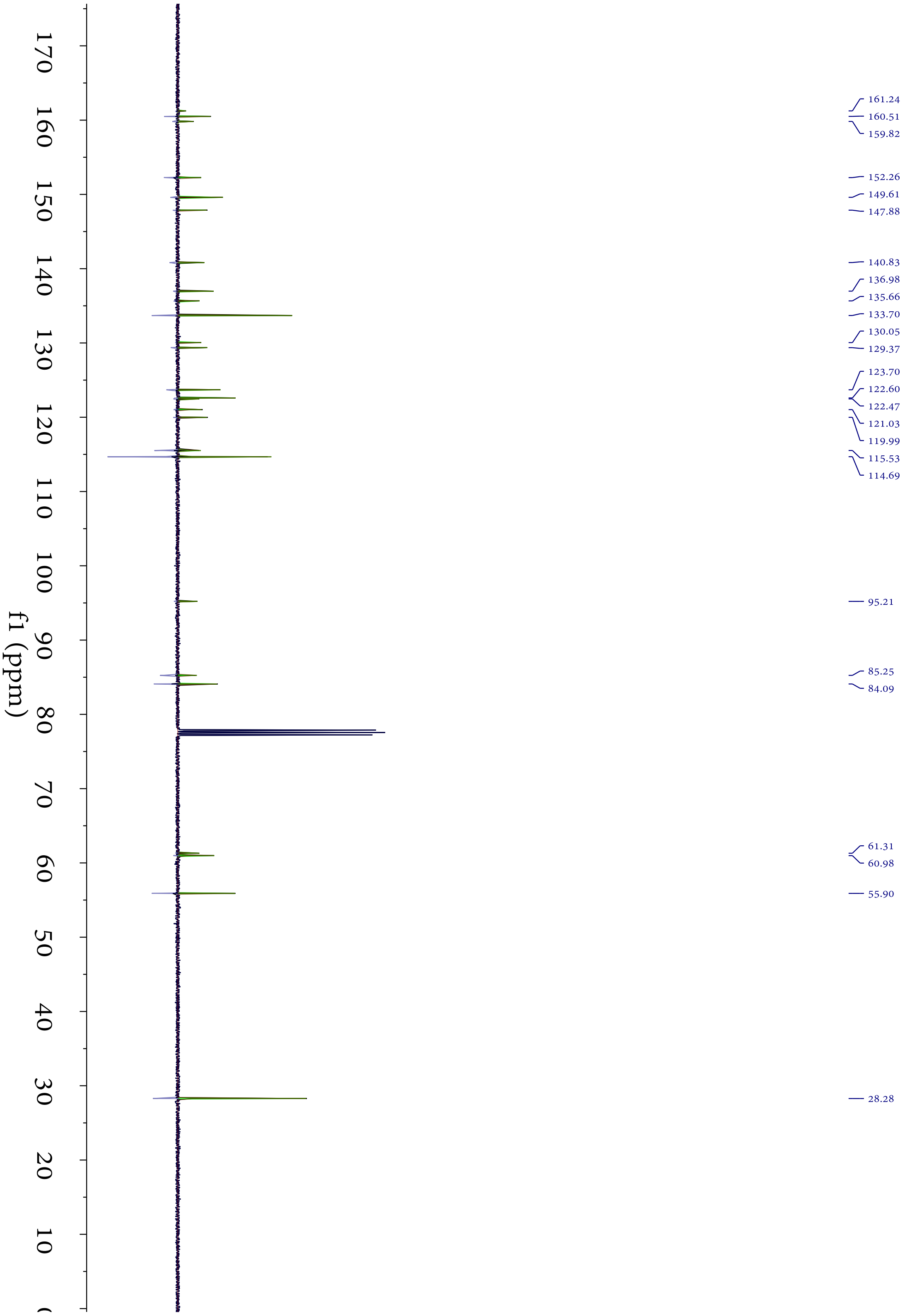

Figure S23. ^{13}C NMR spectrum of **7a** recorded in $CDCl_3$

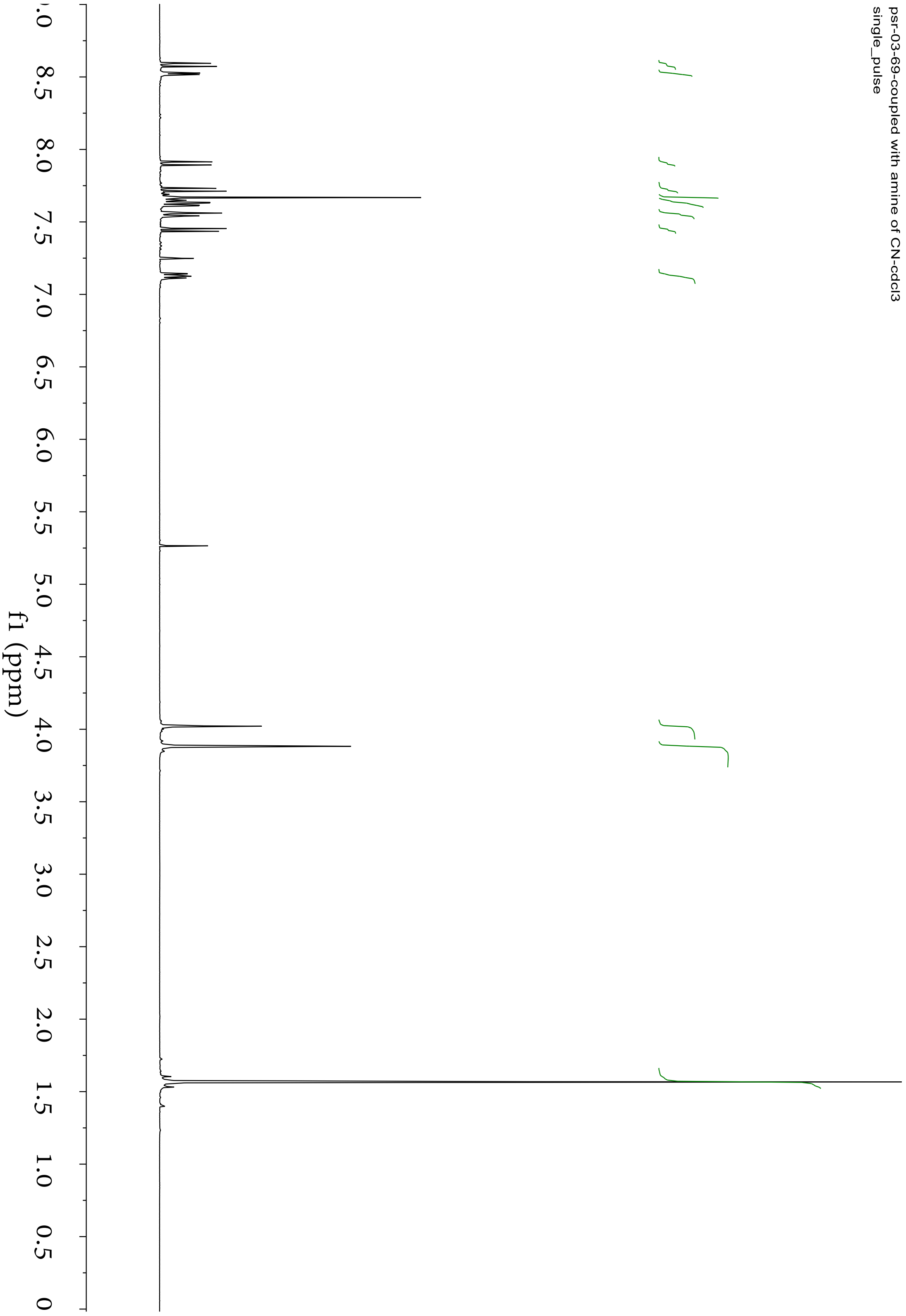

Figure S24. ^{1}H NMR spectrum of **7b** recorded in $CDCl_3$

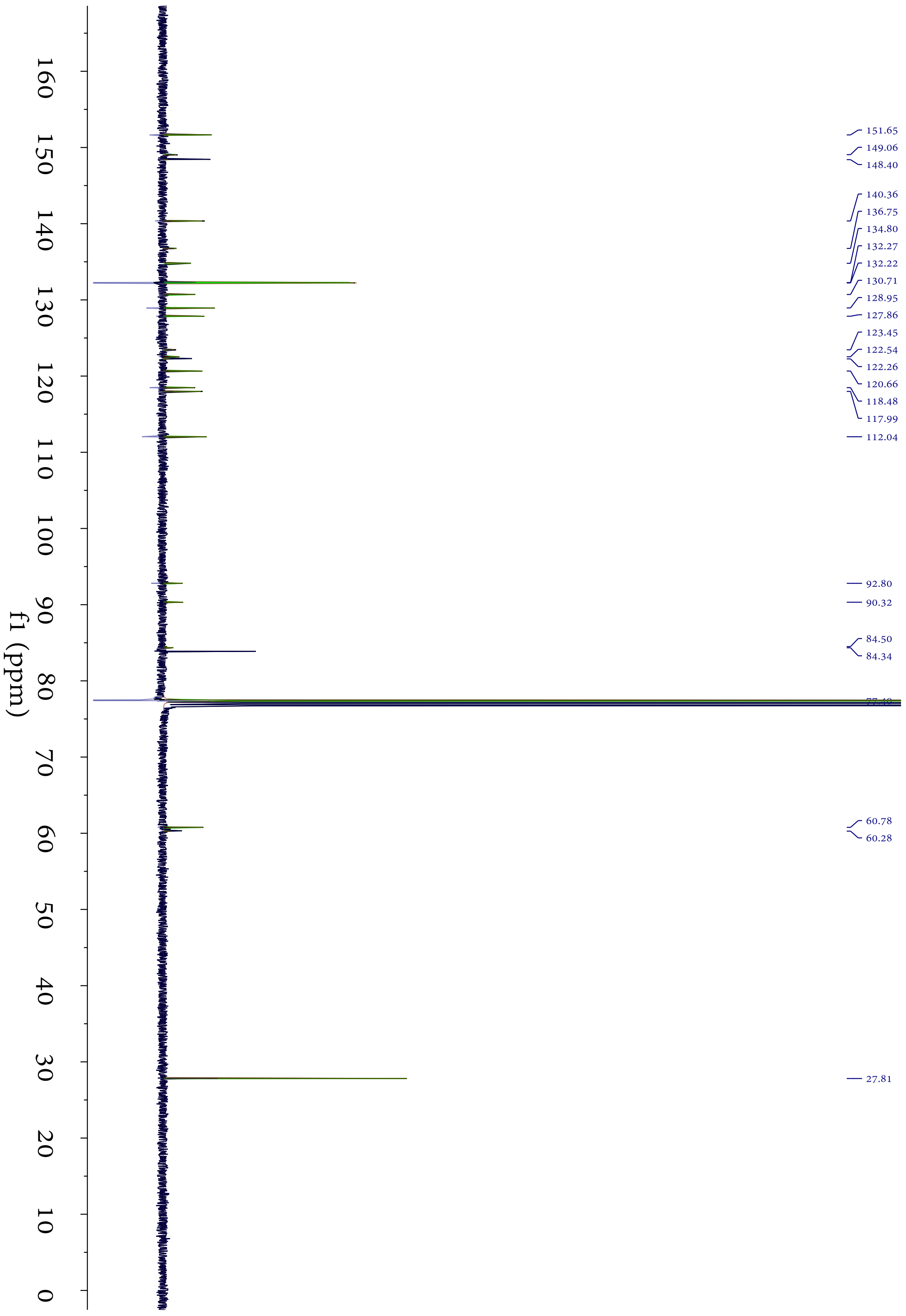

Figure S25. ^{13}C NMR spectrum of **7b** recorded in $CDCl_3$

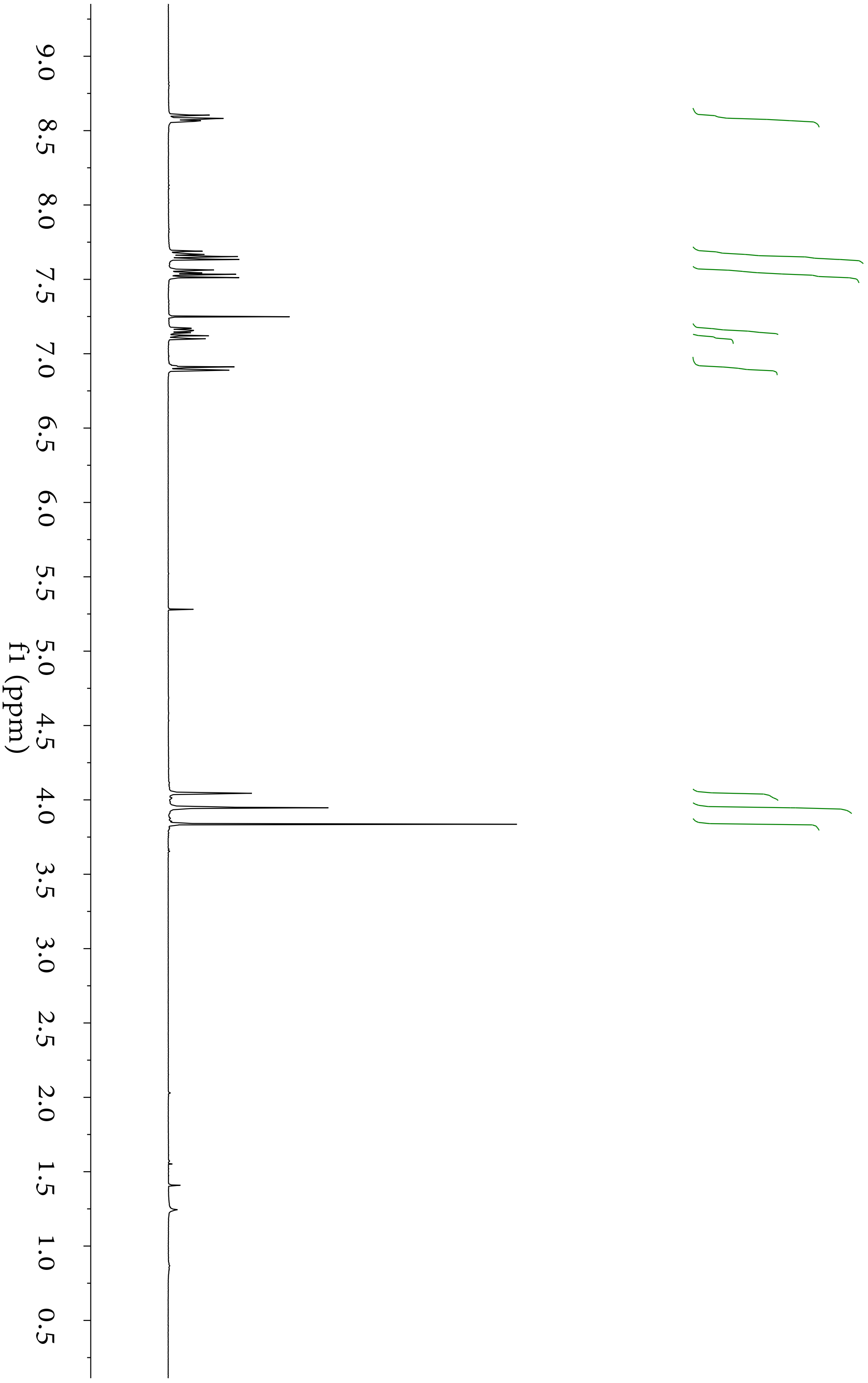

Figure S26. ^{1}H NMR spectrum of **2a** recorded in $CDCl_3$

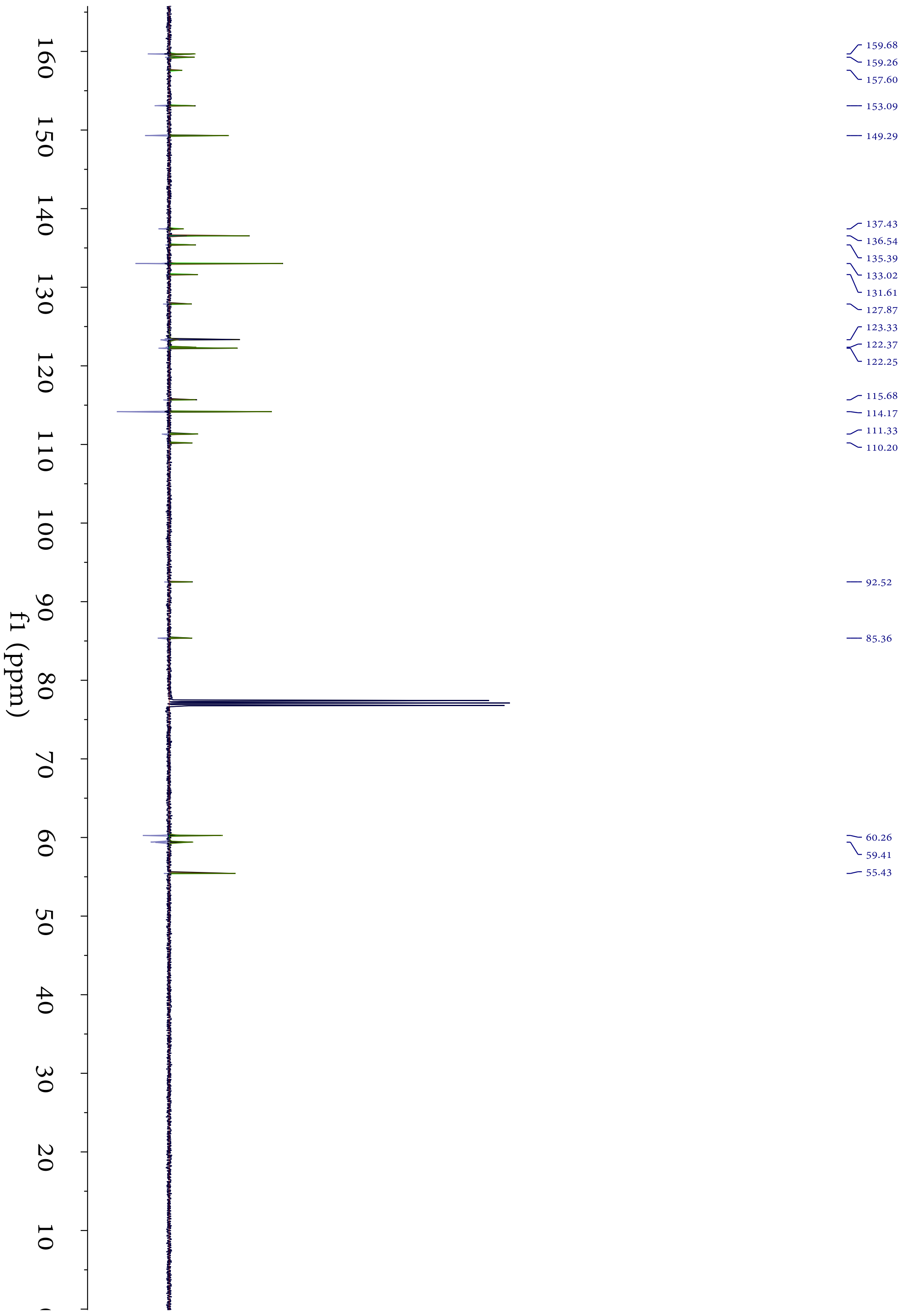

Figure S27. ^{13}C NMR spectrum of **2a** recorded in $CDCl_3$

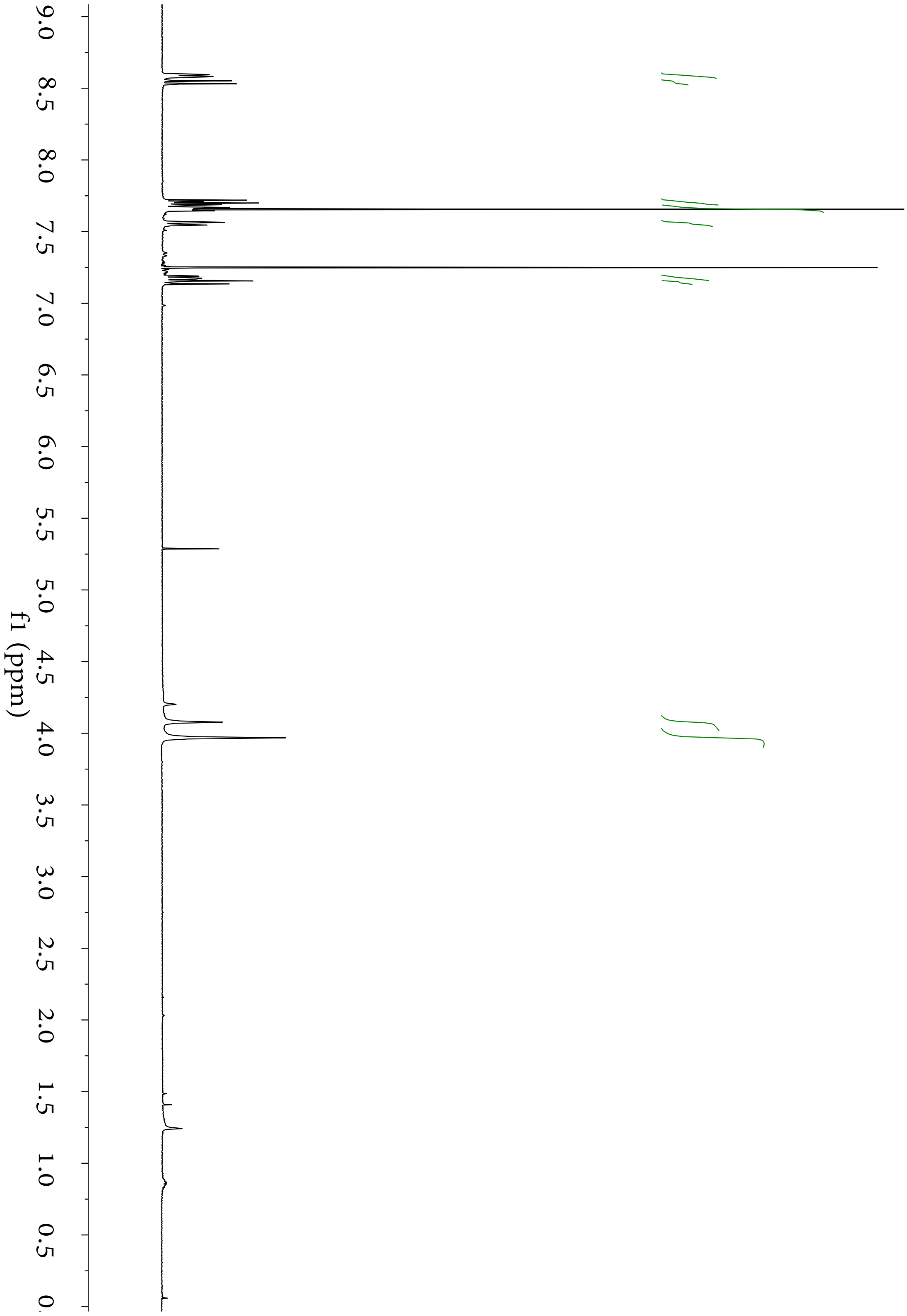

Figure S28. ^{1}H NMR spectrum of **2b** recorded in $CDCl_3$

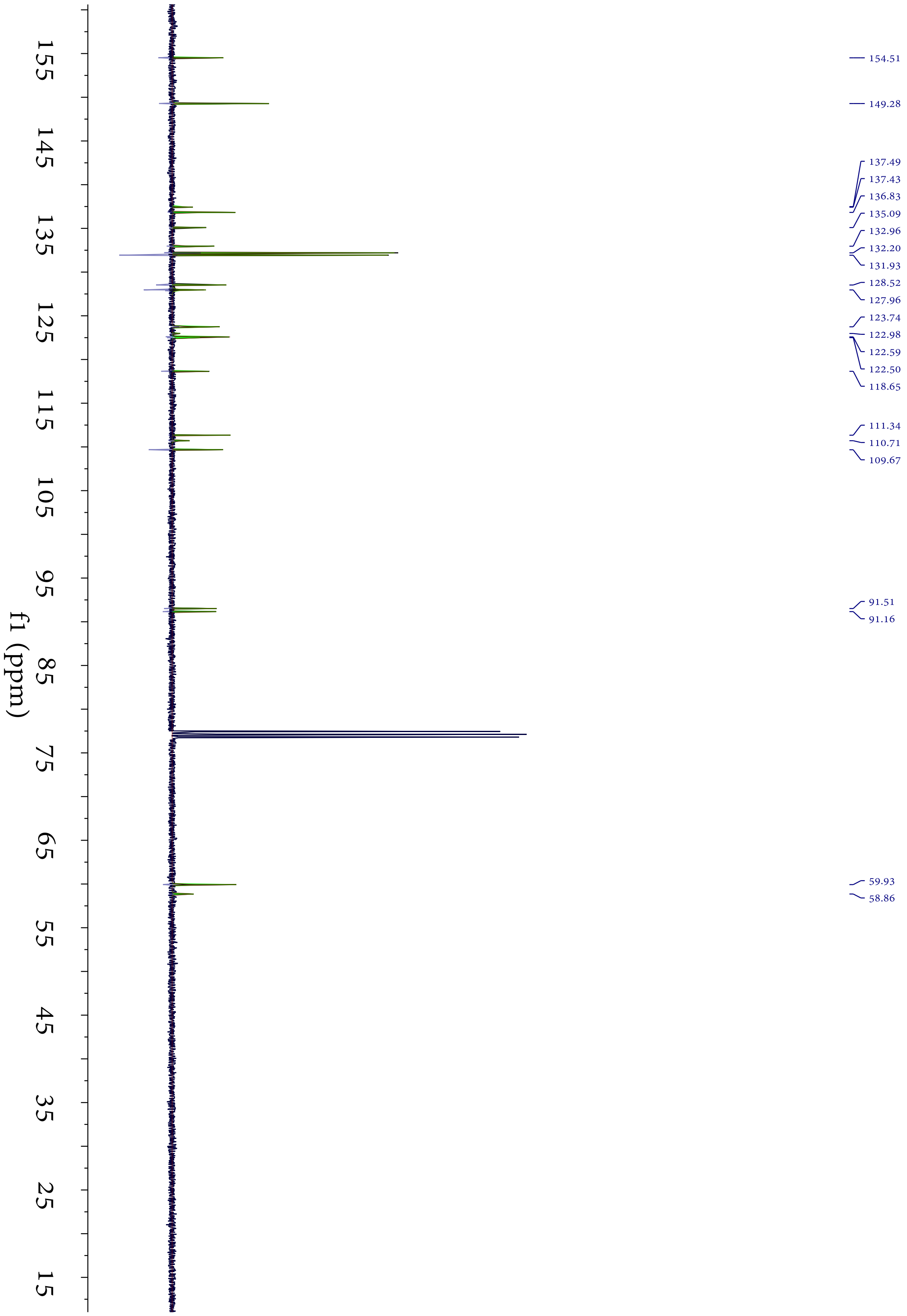

Figure S29. ^{13}C NMR spectrum of **2b** recorded in $CDCl_3$
